# Immune genes are associated with human glioblastoma pathology and patient survival

**DOI:** 10.1186/1755-8794-5-41

**Published:** 2012-09-14

**Authors:** Elodie Vauléon, Avril Tony, Abderrahmane Hamlat, Amandine Etcheverry, Dan Cristian Chiforeanu, Philippe Menei, Jean Mosser, Véronique Quillien, Marc Aubry

**Affiliations:** 1 Department of Medical Oncology, Eugène Marquis Cancer Institute, rue de la bataille Flandres Dunkerque, Rennes 35042, France; 2 CNRS UMR 6061 Genetic and Development, University of Rennes 1, Rennes, France; 3 Department of Clinical Biology, Eugène Marquis Cancer Institute, Rennes, France; 4 Department of Neurosurgery, University Hospital Rennes, Rennes, France; 5 Medical Genomics Unit, Molecular Genetics and Genomics, University Hospital Rennes, Rennes, France; 6 Biogenouest® Genomics Health Platform, University of Rennes 1, Rennes, France; 7 Department of Pathology, University Hospital Rennes, Rennes, France; 8 Department of Neurosurgery, University Hospital Angers, Angers, France

**Keywords:** Glioblastoma, Immune system, Survival

## Abstract

**Background:**

Glioblastoma (GBM) is the most common and lethal primary brain tumor in adults. Several recent transcriptomic studies in GBM have identified different signatures involving immune genes associated with GBM pathology, overall survival (OS) or response to treatment.

**Methods:**

In order to clarify the immune signatures found in GBM, we performed a co-expression network analysis that grouped 791 immune-associated genes (IA genes) in large clusters using a combined dataset of 161 GBM specimens from published databases. We next studied IA genes associated with patient survival using 3 different statistical methods. We then developed a 6-IA gene risk predictor which stratified patients into two groups with statistically significantly different survivals. We validated this risk predictor on two other Affymetrix data series, on a local Agilent data series, and using RT-Q-PCR on a local series of GBM patients treated by standard chemo-radiation therapy.

**Results:**

The co-expression network analysis of the immune genes disclosed 6 powerful modules identifying innate immune system and natural killer cells, myeloid cells and cytokine signatures. Two of these modules were significantly enriched in genes associated with OS. We also found 108 IA genes linked to the immune system significantly associated with OS in GBM patients. The 6-IA gene risk predictor successfully distinguished two groups of GBM patients with significantly different survival (OS low risk: 22.3 months versus high risk: 7.3 months; p < 0.001). Patients with significantly different OS could even be identified among those with known good prognosis (methylated MGMT promoter-bearing tumor) using Agilent (OS 25 versus 8.1 months; p < 0.01) and RT-PCR (OS 21.8 versus 13.9 months; p < 0.05) technologies. Interestingly, the 6-IA gene risk could also distinguish proneural GBM subtypes.

**Conclusions:**

This study demonstrates the immune signatures found in previous GBM genomic analyses and suggests the involvement of immune cells in GBM biology. The robust 6-IA gene risk predictor should be helpful in establishing prognosis in GBM patients, in particular in those with a proneural GBM subtype, and even in the well-known good prognosis group of patients with methylated MGMT promoter-bearing tumors.

## Background

Glioblastoma multiforme (GBM) is the most common and aggressive primary brain tumor in adults. Despite recent advances in multimodal therapy, prognosis remains limited [1]. Conventional treatment, generally maximal safe surgical resection followed by combination radiation and chemotherapy with temozolomide, fails to prevent tumor recurrence.

Recently, molecular subtypes of brain tumors have been characterized by microarray gene expression profiles [2-6]. These subgroups have been associated with significant differences in tumor aggressiveness, progression, and/or prognosis [7]. Gene expression analysis has been reported as being more accurate than conventional histology [8,9]. Due to this greater accuracy, expression-based classifications offer an opportunity to improve molecular classification of gliomas [6,7] and clinical diagnosis of glioblastomas [2]. Such advances could be helpful in designing future therapeutic trials [4,10].

Many arguments have supported a link between the immune system and glioma pathogenesis. In several epidemiologic studies, glioma incidence is inversely associated with allergy history [11-13]. T-lymphocyte infiltration has been reported in certain glioma patients and an elevated number of intratumoral effector T cells has been recently correlated with a better survival in GBM patients [14].

Interestingly, several transcriptomic studies using microarray technologies have also reported an immune signature in gene expression profiling of glioma [8,10,15,16] and GBM [17-20]. A signature associated with myeloid/macrophagic cells has been reported in most of these studies [10,15,16,18,20], a finding consistent with the known macrophage/microglia infiltration in GBM [21-23]. More recently, transcriptomic studies in glioma have revealed different signatures involving immune genes associated with overall survival (OS) [8,10,15,19]. Gravendeel et al. reported an immune response signature associated with poor survival in glioma (Cluster 23 – the M function category) [8]. Murat et al. reported better outcome in patients with gene clusters characterizing features of innate immune response and macrophages (G24 cluster – 134 probes, among them probes for *CD11b* and *CD163* genes) [19]. In contrast, Irliev et al. found an immune module (M7 module) associated with short survival that includes 449 genes, among them T-cell markers (CD4, CD8) and myeloid markers (MHC class II, TLR1 and TLR2) [15]. An NK cell signature (G12 gene cluster including Fc gamma receptors and DAP-12) has previously been reported in one study with higher level expression in primary GBM with shorter survival compared to low grade astrocytomas and secondary GBM [10].

In order to clarify the possible role of immune cells in GBM pathology and OS, we have performed a co-expression network analysis focusing on 791 genes linked to the immune system. Using a meta-analysis approach and independent validation cohorts, we identified an immune signature of GBM linked to innate immunity involving myeloid and NK cells as well as a 6-immune genes risk-model stratifying patients into two groups with significantly different OS.

## Methods

### Immune-associated (IA) genes

**Figure 1:**
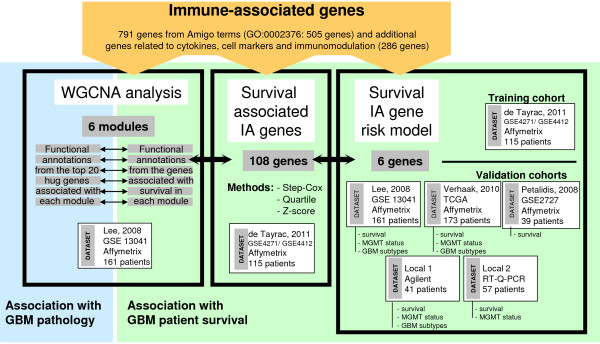
**Analysis workflow.** 791 IA genes were studied in three analyses: weighted gene co-expression network analysis (WGCNA) and functional annotation were performed on the GSE13041 data set (blue box); 108 survival associated IA genes were found by 3 different methods (Step-Cox, Quartile, Z-score) on de Tayrac dataset (middle green box); survival IA gene risk model was built on de Tayrac dataset and validated on 5 datasets: GSE 13041, TCGA, GSE2727, a local Agilent dataset, a local RT-Q- PCR dataset (right hand-side green box).

Immune-associated genes were defined as genes annotated with the ‘immune system process’ Gene Ontology (GO) biological process term (GO:0002376) by the AmiGO annotation tool (505 genes). Important immune-associated genes not annotated with GO:0002376 in GO, such as cytokines, cells markers and immunomodulation genes (286 genes), were added to this GO genes list. This IA genes list is composed of 791 genes (Figure 1) (Additional file 1: Table S1).

### Patients and datasets

For the survival analysis we used four publicly available Affymetrix technology independent microarray datasets (Figure 1) [2,5,7,24]. Moreover, a local cohort including 41 patients with newly diagnosed grade IV glioma admitted to the neurosurgery department of Rennes and Angers University Hospitals was analyzed using a different technology (Agilent). Eventually, a local cohort of 57 newly diagnosed GBM patients, admitted to the neurosurgery department of Rennes University Hospital and homogeneously treated by surgery and radio-chemotherapy with temozolomide like Stupp’s schedule, was analyzed by a reverse transcriptase quantitative polymerase chain reaction (Q-PCR). All patients of the local cohort signed their informed consent. All cohorts and patients characteristics are detailed in Table 1.

**Table 1:** Characteristic of patients and datasets

**Name Dataset origin**		**de Tayrac GSE4271 GSE4412**	**Lee GSE13041**	**Verhaak TCGA Data Portal**	**Petalidis GSE2727**	**Local_1 CHU Rennes CHU Angers**	**Local_2 CHU Rennes**
**Technology Number of samples**		**Affymetrix**	**Affymetrix**	**Affymetrix**	**Affymetrix**	**Agilent**	**Q-PCR**
		**115**	**161**	**173**	**39**	**41**	**57**
Gender	Male	M:65	M:96	M:112	M:29	M:21	M:31
	Female	F:50	F:65	F:61	F:10	F:20	F:26
Age (y)	median [min-max]	48 [18-82]	55 [22-86]	59 [14-87]	61 [22-74]	58 [33-80]	59 [36-78]
	<50	65	59	56	14	10	10
	> = 50	50	102	117	25	31	47
KPS (%)	median	-	-	90 [40-100]	-	80 [40-100]	80 [40-100]
	<=70	-	-	22	-	15	28
	>70	-	-	58	-	23	29
	NA	-	-		-	3	-
Treatment (surgical)	biopsy	-	-	6		1	2
	partial resection	-	-	-		8	17
	total resection	-	161(a)	164	(c)	26	38
	NA	-	-	1		6	-
Treatment (adjuvant)	RT	-				3	-
	RT + CT like Stupp’s schedule	-	(b)	(b)	(c)	37	57
	no treatment	-				1	-
MGMT status	Methylated	-	86	42	-	24	27
	Un-methylated	-	75	122	-	17	29
	NA	-	-	-	-	-	1
IDH1 status	Wild-type	-	-	167	-	39	39
	Mutated	-	-	6	-	2	1
	NA	-	-	-	-	-	17
Subtype	Proneural	-	41	48	-	12	-
	non-Proneural	-	120	125	-	29	-

^(a)^ ‘resection mentioned alone with no other details.

^(b)^ Excessively heterogeneous treatment. GBM *de novo* tumors with no prior treatment.

^(c)^ Data not available.

The MGMT status of the local cohort was obtained by pyrosequencing methylation assay with a threshold of CpG methylation set to ≥9% [25,26]. Local tumor subtypes were determined using the centroid-based classification algorithm described by Verhaak et al. [7].

### Weighted gene co-expression network analysis (WGCNA)

Signed weighted gene co-expression network analysis was performed on the GSE13041 data set [24] (Figure 1 and Table 1). A co-expression network was constructed on the basis of the IA genes. For all possible pairs of the variable genes, Pearson correlation coefficients were calculated across all samples. The correlations matrix was raised to the power 6, thus producing a weighted network. The weighted network was transformed into a network of topological overlap (TO) — an advanced co-expression measure that considers not only the correlation of 2 genes with each other, but also the extent of their shared correlations across the weighted network. Genes were hierarchically clustered on the basis of their TO. Modules were identified on the dendrogram using the Dynamic Tree Cut algorithm [27]. Each gene’s connectivity was determined within its module of residence by summing up the TOs of the gene with all the other genes in the module. By definition, highly connected (hub) genes display expression profiles highly characteristic for their module of residence [28]. To define a measure of prognostic significance, a univariate Cox proportional hazards regression model was used to regress patient survival on the individual gene expression profiles. The resulting p-values were used to define a measure of prognostic significance. To obtain a condensed representative profile of each module, focus was placed on the top 20 hub genes in the module. Co-expression network analyses were performed using the *WGCNA* R package. Survival analyses were performed using the *survival* R package.

### WGCNA modules functional annotation and enrichment

Functional annotation of the IA genes co-expression modules was performed on the basis of the analysis of their top 20 hub genes and survival associated genes in each module. DAVID software ( http://david.abcc.ncifcrf.gov/) was used to test each module for genome enrichment in GO process terms, PIR superfamily, Panther or Kegg pathways, InterPro or SwissProt keywords, and to test IA genes having an impact on overall survival (Fisher’s exact tests with Benjamini-Hochberg correction for multiple testing).

### IA genes associated with patient outcome

Molecular screening of IA genes was performed on 115 GBM patients included in a whole-genome Affymetrix meta-analysis dataset described by de Tayrac et al. [2]. Association between expression levels and patient outcome defined IA genes having an impact on overall survival (OS). Several survival analysis methods were used to identify relevant associations: (i) a Cox-step method [29], (ii) a differential analysis between the first and the fourth quartile, (iii) a classical Cox analysis (Figure 1). Adjusted p-values were calculated by controlling for the false discovery rate with the Benjamini-Hochberg correction. Overall survival was estimated by the Kaplan Meier method. Comparisons between survival groups were performed by the log-rank test. Univariate cox analyses were performed with gene expression data as a predictor and overall survival in months as the response.

### IA genes risk model

An optimal survival model was built on IA genes associated with survival as described in de Tayrac et al. [2]. Analyses were performed using *survival, survivalROC* and *rbsurv* R packages. These packages selected survival-associated genes and estimated the regression coefficients of the optimal survival model after adjustment on the study factor. All analyses were stratified on the age.

### Q-PCR procedures

Total RNA was isolated using Rneasy Plus Mini QIAGEN kit from fresh-frozen glioblastoma samples. RNA integrity was confirmed using the Agilent Bioanalyser (RNA 6000 NAno assay kit). cDNA synthesis was obtained by a High capacity cDNA Reverse Transcription kit with Rnase inhibitor (Applied biosystem®). Q-PCR reactions were done with the 7900HT Fast Real-time PCR System using the Applied biosystem® Taq Man FAM-labeled probes for *ACVR2, CD22, MNX1, ARG1, RPS19* and *FGF2*, and the three housekeeping genes: *TBP*, *HPRT1*, *GAPDH*. Liver cells, testis cells, B lymphocytes and U251 cells were used as positive control. The relative amounts of the gene transcripts were determined using the ΔΔCt method, as described by the manufacturer.

## Results

### IA genes co-expression modules

**Figure 2:**
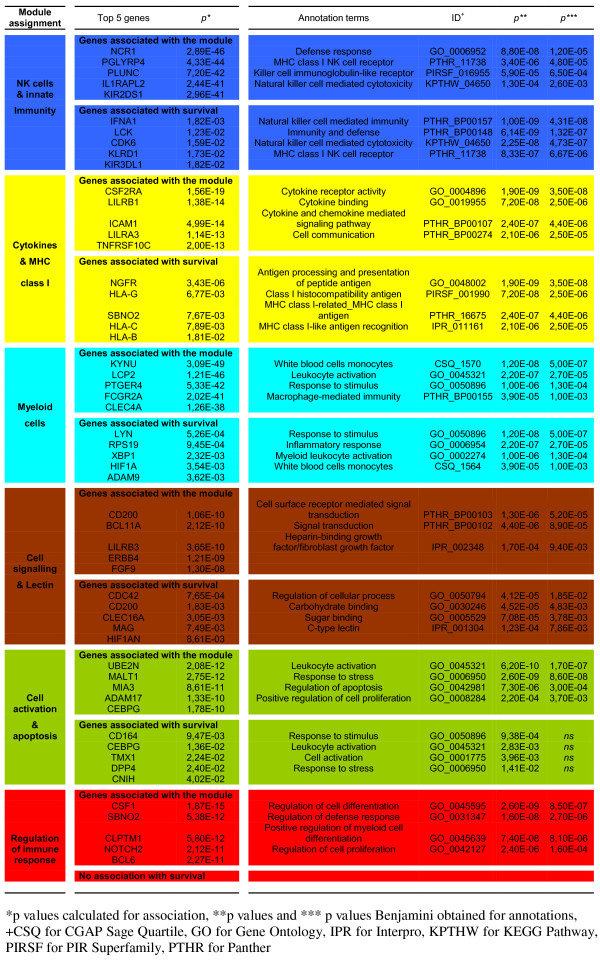
**Gene annotations of the GBM co-expression modules.** Annotations of the top 20 hub genes and survival associated genes in each module were tested for genome enrichment in Gene Ontology process terms (GO), PIR superfamily (PIRSF), Panther (PTHR) or KEGG (KPTHR) pathways, InterPro (IPR) keywords or CGAP Sage tissue expression data (CSQ) using the DAVID program ( http://david.abcc.ncifcrf.gov/). *p values* were calculated for gene association to the module (*) and module annotations (** for *p* and *** Benjamini *p values*).

WGCNA algorithm with the Lee dataset (GSE 13041) was applied to explore transcriptional relationships between IA genes and highlight consistent patterns of gene co-expression [24]. The weighted gene co-expression network constructed on the basis of the IA genes revealed 6 modules, each of them containing coordinately expressed genes potentially involved in shared cellular processes. To associate putative relevant processes and structures with the observed gene co-expression, we analyzed the functional enrichment of each module. For each module, the top five hub IA genes and the first five genes associated with survival are provided in Figure 2. The modules’ annotations were obtained with the top 20 hub IA genes associated with each module and all IA genes associated with survival within this module (Figure 2). The IA genes co-expression modules were thus designated as followed: NK cells and innate immunity (blue module), Cytokines and molecular histocompatibility complex (MHC) class I (yellow module), Myeloid cells (turquoise module), Cell signaling and lectin (brown module), Cell activation and apoptosis (green module) and Regulation of immune response (red module).

### IA genes associated with survival

Interestingly, two co-expression modules were significantly enriched in IA genes having an impact on overall survival: NK cells and innate immunity signature module and the Cytokines and MHC class I signature module (p < 0.01).

**Figure 3:**
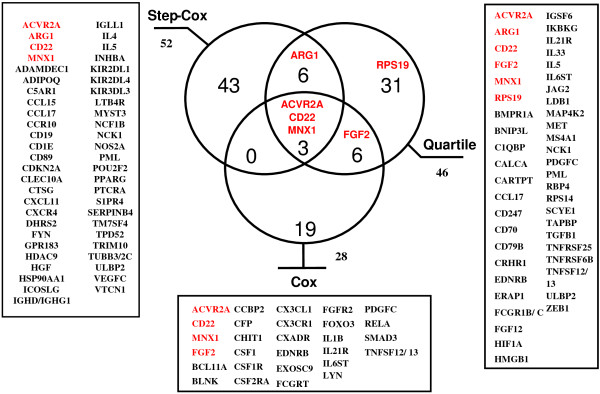
**Venn diagram of IA genes associated with survival in the statistical methods.** 108 survival associated IA genes were found by 3 different methods (Step-Cox, Quartile, Z-score) on de Tayrac dataset. The 6 genes of IA risk model were written in red.

Three different methods were then applied to further analyze the IA genes associated with survival using the de Tayrac dataset. The step-Cox model identified 52 genes associated with overall survival. The quartile model found 46 genes significantly differentially expressed between the lowest survivors and the highest survivors. The classical Cox method identified 28 genes associated with patient outcome (Additional file 1: Table S2). The overlap between the three methods is presented in Figure 3. In conclusion, 108 out of 791 IA genes were found to be associated with GBM patient survival by at least one of the three different statistical methods.

### Risk-score model of IA genes as a GBM outcome predictor

An optimal survival model was built on IA genes associated with survival as described in de Tayrac et al. [2]. The mathematical model included 6 genes: ACVR2A, CD22, MNX1, ARG1, RPS19, FGF2 previously identified as described above. This risk-score equation based on the expression of these 6 genes can be written (0.744 × CD22)+(2.109 × ACVR2A) + (0.860 × MNX1)+ (−1.328 x RPS19) + (−1.028 × FGF2) + (0.913 × ARG1). A risk-score greater than or equal to the threshold of 0.30 signifies a high-risk patient with poor prognosis. Prognosis power is positive with expression of 4 genes (ACVR2A, CD22, MNX1, ARG1) and negative with expression of two others (RPS 19, FGF2).

**Figure 4:**
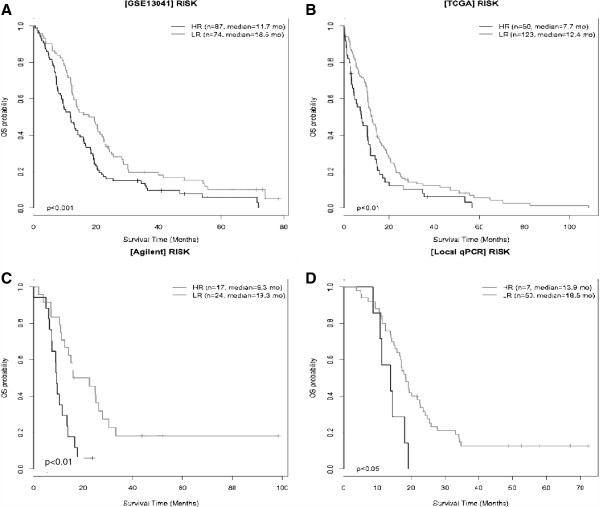
**GBM patient survival according to 6-IA risk.** Kaplan-Meier curves show OS after subdivision into high risk (HR black) and low risk-score groups (LR grey). The median OS was higher in LR than in HR patients from GSE13041 dataset (**A**), TCGA dataset (**B**) Agilent local cohort (**C**) and RT-Q-PCR local cohort (**D**).

The risk-model (threshold = 0.30) stratified the training cohort (de Tayrac dataset) [2] into 2 groups with a significant difference in OS (p = 4.0E-13). The low-risk (n = 66) and high-risk (n = 49) groups had a median OS of 22.3 and 7.3 months, respectively. Stratification of the validation cohort (GSE 2727 published by Petalidis et al. [5]) led to a significant difference of OS (low risk group (n = 18): 12 months versus high risk group (n = 21): 6 months; p = 1.2E-4). The robustness of the 6-IA gene risk-score equation was also checked by using 2 external and publicly available studies performed on Affymetrix technologies (Table 1). The predictor identified two groups of patients with a significant difference in OS using the GSE13041 cohort [24] (p < 0.001) and the TCGA cohort [7] (p < 0.01). Median OS and number of patients in each risk category are provided in Figure 4.

The performance of the 6-IA gene risk model was further tested on a local cohort of 41 patients using Agilent expression microarrays. Low-risk patients had a significantly better survival than high-risk patients (median OS of 19.3 months versus 9.3 months respectively; p < 0.01; Figure 4C). Eventually, reverse transcription Q-PCR based expression measurement of the 6-IA gene risk model genes was performed on a local cohort of 57 patients treated homogenously. Low-risk patients had also a significantly better survival than high-risk patients (median OS of 18.5 months versus 13.9 months respectively; p < 0.05; Figure 4D).

### IA genes risk-score model and MGMT methylation status

**Figure 5:**
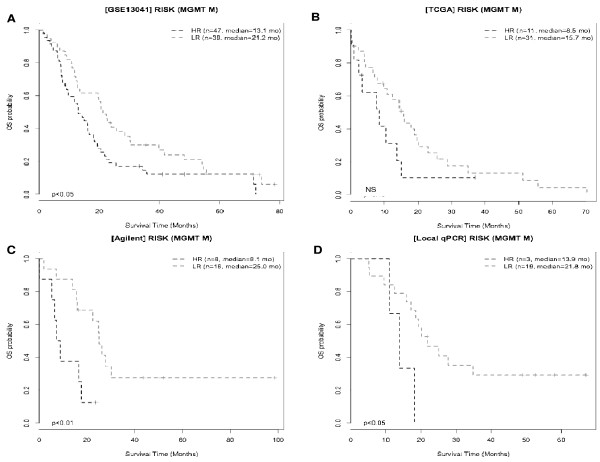
**Survival according to 6-IA risk in patients bearing tumor with methylated MGMT promoter.** Kaplan-Meier curves show OS of patients bearing tumors with methylated MGMT promoters after subdivision into HR (black) and LR (grey) groups. The median OS was higher in LR than in HR patients from GSE13041 dataset (**A**), TCGA dataset (**B**), Agilent local cohort (**C**) and RT-Q-PCR local cohort (**D**).

In univariate Cox analysis using the de Tayrac dataset, the only factors associated with survival were the *MGMT* promoter methylation status and the 6-IA gene risk category. Sex, histology, age and KPS were not statistically associated with patient outcome. In multivariate analysis, the *MGMT* promoter methylation status and the 6-IA gene risk category were still significant (p = 0.02 and p = 0.01, respectively). Difference of survival defined by the 6-IA gene risk remained significant when considering patients bearing tumors with methylated MGMT promoters (25 versus 8.1 months, n = 8 and 16 respectively, p < 0.01; Figure 5C), as in the Lee dataset (21.2 versus 13.1 months, p < 0;05, Figure 5A). In the Q-PCR cohort, the MGMT status and the 6-IA gene risk category were also significantly associated with OS of GBM patients, in both univariate and multivariate analysis (p = 0.045 and p = 0.036, respectively). Nineteen patients with low risk had a median survival of 21.8 months versus 13.9 months in three patients with high risk. Although the number of high-risk patients is low, the difference remains significant (p < 0.05; Figure 5D). No significant difference in survival could be found among patients bearing tumors with methylated MGMT promoters only in the TCGA cohort (Figure 5B). This might be explained by insufficient statistical power, especially since a significant difference was found in the 122 unmethylated MGMT promoter tumors from the TCGA cohort (data not shown).

### IA genes risk-score model and GBM subtypes

**Figure 6:**
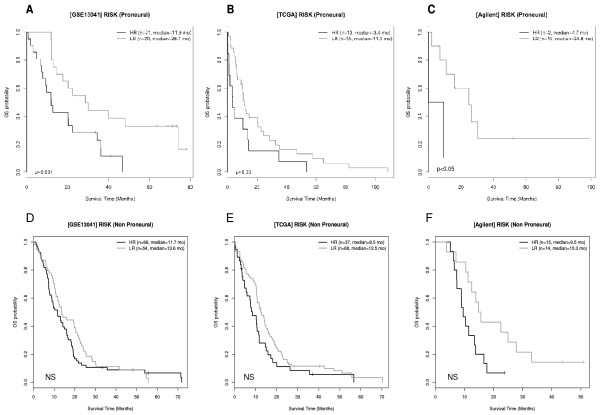
**Survival according to 6-IA risk in patients bearing proneural and non proneural GBM.** Kaplan-Meier curves show OS after subdivision into high (black) and low (LR grey) risk-score groups. The median OS was higher in LR than in HR patients bearing proneural GBM from GSE13041 dataset (**A**), TCGA dataset (**B**), Agilent local cohort (**C**). No significant difference was observed in patients with non proneural GBM from the three datasets (**D**-**F**).

The 6-IA gene risk predictor was also applied to a local cohort and to the cohorts described by Lee and Verhaak [7,24] taking into account the recent GBM classification published by Phillips and Verhaak [6,7]. As only the proneural subtype is associated to survival [24], GBM specimens were divided into two sub-groups: proneural (25% in GSE13041, 38% in TCGA, 29% in the local cohort) and non proneural (Table 1). The 6-IA gene risk predictor classed the patients with proneural GBM into two groups exhibiting significant OS difference: 11.9 versus 28.7 months (p < 0.01; [24]); 11.3 versus 3.4 months (p < 0.05, [7]); 24.8 versus 4.7 months (p < 0.02; in our local cohort) (Figure 6 A-C). Conversely, no difference was observed in the non proneural group of GBM (Figure 6 D-F).

## Discussion

In this study, we were able to link IA genes expression pattern with GBM biology and patient survival. Indeed, our co-expression network analysis highlighted clusters of IA genes and revealed related immune signatures marking innate immunity, NK and myeloid cells and cytokines/MHC class I molecules profiles. Furthermore, 108 IA genes were associated with OS. Among these, 6 IA genes were included in a weighted multigene risk model that can predict outcome in GBM patients.

Several studies have previously reported an immune signature in GBM [8,10,15-17,19,20,30]. A signature associated with myeloid/macrophagic cells was reported in most of these [10,15,16,18,20]. We also found such a signature linked to one co-expression module for which annotation enrichment found monocytes, leukocyte activation and macrophage-mediated immunity. The well known macrophage/microglia infiltration in GBM can account for up to one-third of cells in some GBM specimens [21-23]. Unlike Ivliev et al. [15], we were unable to identify a T-cell signature in our analysis. Nevertheless, the association of two gene modules with GBM patient survival suggests that innate immunity including NK cell functions and cytokines/CMH class I profiles might affect outcome in GBM patients. A NK cell signature has previously been reported in one study in primary GBM [10]. NK cell infiltration was described earlier in glioma [31] but was not confirmed by others [32]. It is noteworthy that in murine glioma models, various vaccines strategies using CCL2 [33], CpG [34], IL12-expressing stroma cells [35] or IL23-expressing dendritic cells [36], induced an increased recruitment of NK cells at the tumor site, associated with better overall survival.

Most of chemokines present in the cytokines/MHC class I module are involved in recruiting T cells, monocytes/macrophages and neutrophils: e.g. *CX3CR1/CX3CL1*, *CXCL9* and *CXCR2* genes. In addition, most of the cytokines found such as *MIF*, *IL5*, *IL12A* and *IL16* genes are known to regulate macrophages/monocytes, eosinophils, NK and T cells. Lohr has also reported that intratumoral infiltration of effector T cells is associated with a better survival in GBM [14]. In total, one could speculate that these two modules associated with overall survival reflect the recruitment and activation of immune cells such as NK cell, T cell, macrophages/monocytes, or neutrophils that would interfere with GBM patients’ survival. Interestingly, several clinical trials using dendritic cells have reported that the presence of T cells and neutrophils at the tumor site is associated with longer survival of the vaccinated patients [37]. Recently, Ducray et al. reported that infiltration of both CD3+ T cells and CD68+ macrophages was observed more frequently in GBM responders than in non-responders to radiotherapy [17]. However, in the present study, we did not find any association between key regulators of the T cell biology such as *GATA3*, *TBX21* (*TBET*), and *RORC* (*ROR-gamma-t*) with patients’ survival (data not shown). The small amount of these infiltrating cells is usually reported in the GBM specimens and might have impaired the identification of such genes by a transcriptomic approach.

In addition to the co-expression network analysis, we have found 108 IA genes directly associated with OS in GBM patient using three different statistical methods. These genes are known to be involved in the biology of B cells (i.e. *immunoglobulins*, *BLNK*, *CD19*, *CD20* and *CD22* genes), T cells (i.e. *CD1E*, *PTCRA*, *CD247*), NK cells (i.e. *KIR2DL1*, *KIR2DL4* and *KIR3DL3* genes), and myeloid cells including monocytes/macrophages (i.e. *ADAMDEC1*, *CD89/FCAR*, *CD64*/*FCGR1B* and *FCGR1C* genes) and neutrophils (i.e. *CD89*, and *NCF1B* genes). Surprisingly, other important genes expressed by glioma-infiltrating microglia/macrophages, such as *CD163* and *AIF1* (*IBA1*), were not significantly associated with patients’ survival (data not shown). Komohara et al. have recently reported that the presence of CD163+ CD204+ M2-type macrophagic cells correlates with glioma grading and survival using an immunohistochemistry approach [38]. This discrepancy between our results and the Komohara et al. study could be explained by the fact that we used different technical approaches to detect these markers: at the mRNA level in our genomic study and at the protein level in [38]. Others genes of chemokines and cytokines have been also found such as *CCL15*, *CCL17 IL1B* and *IL5* genes. Finally, some genes are known to be involved in the modulation/suppression of the immune response such as *APRIL*, *ARG1*, *CD70*, *B7-H4*, *ICOSLG*, *NOS2A*, *TGFB1* and *TWEAK* genes.

Finally, we have developed a 6-IA-gene risk predictor of OS in GBM patients. The genes have been selected for an optimal survival model built on IA genes associated with survival as described in de Tayrac et al. [2]. This 6-IA gene risk is able to discriminate patients treated by chemo-radiation therapy into two distinct groups with significantly different survivals. These genes *ACVR2A*, *ARG1*, *CD22*, *FGF2*, *MNX1* and *RPS19* were present in all but one of the co-expression modules. The ‘regulation of immune response’ module, which contains no gene retained in the 6-IA-gene risk predictor, is the only one that does not include survival-associated genes. *ACVR2A*, *CD22* and *MNX1* genes were found to be associated with GBM patient survival in the three different statistical methods. Intriguingly, these 6 IA genes are not specific markers for known immune cell subpopulations. They are involved in the activation or the inhibition of the immune system. As a result, they impact positively or negatively on the risk predictor. For example, the expression of ARG1, a gene involved in immunosuppression, contributes positively to the 6-IA-gene risk index and therefore decreases the patient’s probability of survival. Although these genes are known in other cancers, they have not been described in GBM. ACVR2A is a receptor for activin-A and controls cell proliferation [39], for example proliferation of prostate cancer cells [40]. Mutations of ACVR2A are commonly found in unstable colonic cancers [41], and interestingly, infiltration of CD3 T cells is associated with mutated *ACVR2A* genes [42]. ARG1 for arginase-1 is a cytosolic enzyme that hydrolyses arginine to urea and ornithine [43]. ARG1 has recently been involved in immunosuppressive mechanisms by reducing T-cell activation [44]. CD22 cannot be considered only to be a B cell receptor that mediates cell adhesion and signaling [45,46] since Mott et al. report that neurons can secrete this molecule [47]. Neuronal secretion of CD22 inhibits microglia activation via interaction with CD45 [47]. FGF2 for fibroblast growth factor-2 stimulates GBM growth [48]. Nevertheless, the high molecular weight FGF2 isoform inhibits glioma proliferation [49] and explains the radiation therapy resistance pathway [50]. Interestingly, plasma levels of FGF are higher in GBM patients compared to control [51]. *MNX1* gene is involved in a congenital malformation, the Currarino syndrome (congenital malformation) [52] and also previously reported in CD34+ cells, B cells and B lymphoid tissues [53]. MNX1 function in immune cells and GBM biology has not been demonstrated yet but it has recently been described as a transcriptional factor implicated in the development of both solid and hematological cancers [54]. RPS19 is a subunit of 40S ribosome involved in pre-rRNA processing but also has extra-ribosomal functions. Indeed, RPS19 can act as a chemokine that regulates macrophage migration inhibitory factor (MIF) negatively [55]. Moreover, RPS19 can interact with FGF2 to drive differentiation or proliferation pathways of various cell types [56]. Only one statistical method, the quartile method, found this gene significantly (Figure 3), but the co-expression module found it to be significantly associated with OS (Figure 2).

To validate the strength of our 6-IA-gene risk predictor, expression of these genes was tested in a local cohort using RT Q-PCR. This technique has at least two advantages, it is used routinely in most laboratories and is relatively inexpensive compared with genomic microarray technologies. The test cohort was small (57 GBM specimens) but homogeneous in terms of treatment: combined surgery and chemo-radiation therapy [1]. In addition, the MGMT methylation status, which is the best predictor of response to the current combination treatment, was determined for all GBM specimens. Applied to this small cohort, 6-IA-gene risk predictor was even able to discriminate significantly between patients with high and low risk in the good prognosis group, defined by methylation of the MGMT promoter.

Recent advances in glioma classification have been achieved using genomic analysis. It is now accepted that GBM can be categorized in four subtypes defined as proneural, neural, mesenchymal, and classical groups [6,7,24]. The clinical outcome of the patients is different according to the GBM subtype. For instance, patients with proneural subtype live longer and the standard treatment does not increase their overall survival [6,7]. In contrast, overall survival of patients with classical or mesenchymal subtype is significantly increased with the standard treatment. Interestingly, we have shown that our 6-IA-gene risk predictor was powerful in GBM proneural subtype but not in others subtypes. GBM proneural is an atypical GBM subtype which is associated with younger age, *PDGFRA* gene amplification, *IDH1* mutations, *TP53* mutations [7]. Due to the fact that these patients with proneural GBM have longer survival, one could speculate that the anti-tumor immune response could have more time to occur and slow down the tumor progression in some of these patients with a particular immune profile, revealed by our 6-AI-gene risk predictor.

## Conclusions

In conclusion, we have demonstrated that GBM are characterized by an immune signature which could reflect the infiltration and activation of immune cells or the immunosuppression mechanisms developed by the tumor itself. Several IA genes were found to be associated with clinical outcome of GBM patients, allowing us to describe a 6-IA-gene risk predictor. This risk model can discriminate between patients with different outcomes, even within the good prognosis group based on MGMT status and within the proneural GBM subtype group. Further studies are needed to understand how these IA genes are involved in the control of GBM progression. Overall, this study highlights the important role of the immune system in the battle against the tumor and suggests new strategies for further development of immunotherapy for GBM patients.

## Competing interests

No potential conflicts of interest were disclosed.

## Authors’ contributions

EV carried out the molecular studies, performed the statistical analysis, made the interpretation of the data and drafted the manuscript; TA participated in the design of the study, helped to draft the manuscript; and made the interpretation of the data; AH and PM provided GBM specimens and participated to the draft of manuscript; AE carried out the Agilent local microarray assays and helped to draft the manuscript; DCC reviewed the histopathology of GBM specimens; JM and VQ contributed to the conception and the design of the study and revised critically the manuscript; MA performed the statistical analysis, made the interpretation of the data, contributed to the conception and the design of the study and revised critically the manuscript. All authors read and approved the final manuscript.

## Grant support

This work was supported by Gefluc Rennes Bretagne (Groupement des Entreprises Françaises dans la Lutte contre le Cancer) et la Région Bretagne (CPER «  Biothérapies » ).

## Pre-publication history

The pre-publication history for this paper can be accessed here:

http://www.biomedcentral.com/1755-8794/5/41/prepub

## Supplementary Material

Additional file 1 **Table S1.** List of IA genes. **Table S2.** IA genes associated with survival in the 3 statistical methods. Click here for file
