# Supplementary material for: Immune genes are associated with human glioblastoma pathology and patient survival
Source: BMC Med Genomics. 2012 Sep 14;5:41. doi: 10.1186/1755-8794-5-41 (PMC3507656; doi:10.1186/1755-8794-5-41)
Supplement: Additional file 1 — Table S1. List of IA genes. Table S2. IA genes associated with survival in the 3 statistical methods. [file 1755-8794-5-41-S1.pdf]

| | Gene Name | UniGene ID | Gene ID | Description |
|---|---|---|---|---|
| 1 | ABCC9 | Hs.446050 | 10060 | ATP-binding cassette, sub-family C (CFTR/MRP), member 9 |
| 2 | ACE | Hs.654434 | 1636 | Angiotensin I converting enzyme (peptidyl-dipeptidase A) 1 |
| 3 | ACVR1B | Hs.438918 | 91 | Activin A receptor, type IB |
| 4 | ACVR2A | Hs.470174 | 92 | Activin A receptor, type IIA |
| 5 | ADA | Hs.654536 | 100 | Adenosine deaminase |
| 6 | ADAM10 | Hs.578508 | 102 | ADAM metallopeptidase domain 10 |
| 7 | ADAM17 | Hs.404914 | 6868 | ADAM metallopeptidase domain 17 (tumor necrosis factor, alpha, converting enzyme) |
| 8 | ADAM19 | Hs.483944 | 8728 | ADAM metallopeptidase domain 19 (meltrin beta) |
| 9 | ADAM9 | Hs.591852 | 8754 | ADAM metallopeptidase domain 9 (meltrin gamma) |
| 10 | ADAMDEC1 | Hs.521459 | 27299 | ADAM-like, decysin 1 |
| 11 | ADIPOQ | Hs.80485 | 9370 | Adiponectin, C1Q and collagen domain containing |
| 12 | ADSS | Hs.498313 | 159 | Adenylosuccinate synthase |
| 13 | AICDA | Hs.149342 | 57379 | Activation-induced cytidine deaminase |
| 14 | AIM2 | Hs.281898 | 9447 | Absent in melanoma 2 |
| 15 | AIMP1 | Hs.591680 | 9255 | Aminoacyl tRNA synthase complex-interacting multifunctional protein 1 |
| 16 | AKAP17A | Hs.522572 | 8227 | A-kinase anchor protein 17A |
| 17 | AKT1 | Hs.525622 | 207 | V-akt murine thymoma viral oncogene homolog 1 |
| 18 | ALAS2 | Hs.522666 | 212 | Aminolevulinate, delta-, synthase 2 (sideroblastic/hypochromic anemia) |
| 19 | AMBP | Hs.436911 | 259 | Alpha-1-microglobulin/bikunin precursor |
| 20 | ANXA11 | Hs.530291 | 311 | Annexin A11 |
| 21 | AP2A2 | Hs.19121 | 161 | Adaptor-related protein complex 2, alpha 2 subunit |
| 22 | AP2B1 | Hs.514819 | 163 | Adaptor-related protein complex 2, beta 1 subunit |
| 23 | AP2M1 | Hs.518460 | 1173 | Adaptor-related protein complex 2, mu 1 subunit |
| 24 | AP2S1 | Hs.119591 | 1175 | Adaptor-related protein complex 2, sigma 1 subunit |
| 25 | APOA1 | Hs.633003 | 335 | Apolipoprotein A-I |
| 26 | APOA2 | Hs.237658 | 336 | Apolipoprotein A-II |
| 27 | APOA4 | Hs.591940 | 337 | Apolipoprotein A-IV |
| 28 | APOBEC3F | Hs.659991 | 200316 | Apolipoprotein B mRNA editing enzyme, catalytic polypeptide-like 3F |
| 29 | APOBEC3G | Hs.660143 | 60489 | Apolipoprotein B mRNA editing enzyme, catalytic polypeptide-like 3G |
| 30 | APOL1 | Hs.114309 | 8542 | Apolipoprotein L, 1 |
| 31 | AQP3 | Hs.234642 | 360 | Aquaporin 3 (Gill blood group) |
| 32 | AQP9 | Hs.104624 | 366 | Aquaporin 9 |
| 33 | ARG1 | Hs.440934 | 383 | Arginase, liver |
| 34 | ARG2 | Hs.705408 | 384 | Arginase, type II |
| 35 | ARHGDIB | Hs.504877 | 397 | Rho GDP dissociation inhibitor (GDI) beta |
| 36 | ARNT | Hs.632446 | 405 | Aryl hydrocarbon receptor nuclear translocator |
| 37 | ASH2L | Hs.521530 | 9070 | Ash2 (absent, small, or homeotic)-like (Drosophila) |
| 38 | ATP6V0A2 | Hs.201939 | 23545 | ATPase, H+ transporting, lysosomal V0 subunit a2 |
| 39 | ATP6V1H | Hs.491737 | 51606 | ATPase, H+ transporting, lysosomal 50/57kDa, V1 subunit H |
| 40 | ATP7A | Hs.496414 | 538 | ATPase, Cu++ transporting, alpha polypeptide (Menkes syndrome) |
| 41 | AZU1 | Hs.72885 | 566 | Azurocidin 1 (cationic antimicrobial protein 37) |
| 42 | B9D2 | Hs.567596 | 80776 | B9 protein domain 2 |
| 43 | BCAP31 | Hs.522817 | 10134 | B-cell receptor-associated protein 31 |
| 44 | BCL10 | Hs.193516 | 8915 | B-cell CLL/lymphoma 10 |
| 45 | BCL11A | Hs.370549 | 53335 | B-cell CLL/lymphoma 11A (zinc finger protein) |
| 46 | BCL2 | Hs.150749 | 596 | B-cell CLL/lymphoma 2 |
| 47 | BCL6 | Hs.478588 | 604 | B-cell CLL/lymphoma 6 (zinc finger protein 51) |
| 48 | BGLAP | Hs.654541 | 632 | Bone gamma-carboxyglutamate (gla) protein (osteocalcin) |
| 49 | BLNK | Hs.665244 | 29760 | B-cell linker |
| 50 | BMP6 | Hs.285671 | 654 | Bone morphogenetic protein 6 |
| 51 | BMPR1A | Hs.524477 | 657 | Bone morphogenetic protein receptor, type IA |
| 52 | BNIP3 | Hs.144873 | 664 | BCL2/adenovirus E1B 19kDa interacting protein 3 |
| 53 | BNIP3L | Hs.131226 | 665 | BCL2/adenovirus E1B 19kDa interacting protein 3-like |
| 54 | BPI | Hs.529019 | 671 | Bactericidal/permeability-increasing protein |
| 55 | BST1 | Hs.169998 | 683 | Bone marrow stromal cell antigen 1 |
| 56 | BST2 | Hs.118110 | 684 | Bone marrow stromal cell antigen 2 |
| 57 | C1QBP | Hs.555866 | 708 | Complement component 1, q subcomponent binding protein |

| | | | | |
|---|---|---|---|---|
| 58 | C2 | Hs.408903 | 717 | Complement component 2 |
| 59 | C3 | Hs.529053 | 718 | Complement component 3 |
| 60 | C5AR1 | Hs.2161 | 728 | Complement component 5a receptor 1 |
| 61 | CADM1 | Hs.370510 | 23705 | Cell adhesion molecule 1 |
| 62 | CALCA | Hs.37058 | 796 | Calcitonin/calcitonin-related polypeptide, alpha |
| 63 | CALCOCO2 | Hs.514920 | 10241 | Calcium binding and coiled-coil domain 2 |
| 64 | CALR | Hs.515162 | 811 | Calreticulin |
| 65 | CARTPT | Hs.1707 | 9607 | CART prepropeptide |
| 66 | CCBP2 | Hs.146346 | 1238 | Chemokine binding protein 2 |
| 67 | CCL1 | Hs.72918 | 6346 | Chemokine (C-C motif) ligand 1 |
| 68 | CCL11 | Hs.54460 | 6356 | Chemokine (C-C motif) ligand 11 |
| 69 | CCL13 | Hs.414629 | 6357 | Chemokine (C-C motif) ligand 13 |
| 70 | CCL16 | Hs.10458 | 6360 | Chemokine (C-C motif) ligand 16 |
| 71 | CCL17 | Hs.546294 | 6361 | Chemokine (C-C motif) ligand 17 |
| 72 | CCL18 | Hs.143961 | 6362 | Chemokine (C-C motif) ligand 18 (pulmonary and activation-regulated) |
| 73 | CCL19 | Hs.50002 | 6363 | Chemokine (C-C motif) ligand 19 |
| 74 | CCL2 | Hs.303649 | 6347 | Chemokine (C-C motif) ligand 2 |
| 75 | CCL20 | Hs.75498 | 6364 | Chemokine (C-C motif) ligand 20 |
| 76 | CCL21 | Hs.57907 | 6366 | Chemokine (C-C motif) ligand 21 |
| 77 | CCL22 | Hs.534347 | 6367 | Chemokine (C-C motif) ligand 22 |
| 78 | CCL23 | Hs.169191 | 6368 | Chemokine (C-C motif) ligand 23 |
| 79 | CCL24 | Hs.247838 | 6369 | Chemokine (C-C motif) ligand 24 |
| 80 | CCL25 | Hs.310511 | 6370 | Chemokine (C-C motif) ligand 25 |
| 81 | CCL27 | Hs.459590 | 10850 | Chemokine (C-C motif) ligand 27 |
| 82 | CCL4 | Hs.75703 | 6351 | Chemokine (C-C motif) ligand 4 |
| 83 | CCL5 | Hs.514821 | 6352 | Chemokine (C-C motif) ligand 5 |
| 84 | CCL7 | Hs.251526 | 6354 | Chemokine (C-C motif) ligand 7 |
| 85 | CCL8 | Hs.699804 | 6355 | Chemokine (C-C motif) ligand 8 |
| 86 | CCR1 | Hs.301921 | 1230 | Chemokine (C-C motif) receptor 1 |
| 87 | CCR10 | Hs.703555 | 2826 | Chemokine (C-C motif) receptor 10 |
| 88 | CCR2 | Hs.644637 | 1231 | Chemokine (C-C motif) receptor 2 |
| 89 | CCR3 | Hs.506190 | 1232 | Chemokine (C-C motif) receptor 3 |
| 90 | CCR4 | Hs.184926 | 1233 | Chemokine (C-C motif) receptor 4 |
| 91 | CCR5 | Hs.450802 | 1234 | Chemokine (C-C motif) receptor 5 |
| 92 | CCR6 | Hs.46468 | 1235 | Chemokine (C-C motif) receptor 6 |
| 93 | CCR7 | Hs.370036 | 1236 | Chemokine (C-C motif) receptor 7 |
| 94 | CCR8 | Hs.113222 | 1237 | Chemokine (C-C motif) receptor 8 |
| 95 | CCR9 | Hs.225946 | 10803 | Chemokine (C-C motif) receptor 9 |
| 96 | CCRL1 | Hs.310512 | 51554 | Chemokine (C-C motif) receptor-like 1 |
| 97 | CCRL2 | Hs.535713 | 9034 | Chemokine (C-C motif) receptor-like 2 |
| 98 | CD14 | Hs.163867 | 929 | CD14 molecule |
| 99 | CD163 | Hs.504641 | 9332 | CD163 molecule |
| 100 | CD164 | Hs.520313 | 8763 | CD164 molecule, sialomucin |
| 101 | CD19 | Hs.652262 | 930 | CD19 molecule |
| 102 | CD1A | Hs.1309 | 909 | CD1a molecule |
| 103 | CD1D | Hs.1799 | 912 | CD1d molecule |
| 104 | CD1E | Hs.249217 | 913 | CD1e molecule |
| 105 | CD2 | Hs.523500 | 914 | CD2 molecule |
| 106 | CD200 | Hs.79015 | 4345 | CD200 molecule |
| 107 | CD207 | Hs.199731 | 50489 | CD207 molecule, langerin |
| 108 | CD209 | Hs.278694 | 30835 | CD209 molecule |
| 109 | CD22 | Hs.643440 | 933 | CD22 molecule |
| 110 | CD226 | Hs.660130 | 10666 | CD226 molecule |
| 111 | CD24 | Hs.644105 | 934 | CD24 molecule |
| 112 | CD244 | Hs.157872 | 51744 | CD244 molecule, natural killer cell receptor 2B4 |
| 113 | CD247 | Hs.156445 | 919 | CD247 molecule |
| 114 | CD27 | Hs.355307 | 939 | CD27 molecule |
| 115 | CD28 | Hs.591629 | 940 | CD28 molecule |

| | | | | |
|---|---|---|---|---|
| 116 | CD302 | Hs.130014 | 9936 | CD302 molecule |
| 117 | CD320 | Hs.558499 | 51293 | CD320 molecule |
| 118 | CD33 | Hs.83731 | 945 | CD33 molecule |
| 119 | CD34 | Hs.374990 | 947 | CD34 molecule |
| 120 | CD3D | Hs.504048 | 915 | CD3d molecule, delta (CD3-TCR complex) |
| 121 | CD3E | Hs.3003 | 916 | CD3e molecule, epsilon (CD3-TCR complex) |
| 122 | CD3EAP | Hs.705916 | 10849 | CD3e molecule, epsilon associated protein |
| 123 | CD3G | Hs.2259 | 917 | CD3g molecule, gamma (CD3-TCR complex) |
| 124 | CD4 | Hs.631659 | 920 | CD4 molecule |
| 125 | CD40 | Hs.472860 | 958 | CD40 molecule, TNF receptor superfamily member 5 |
| 126 | CD40LG | Hs.592244 | 959 | CD40 ligand (TNF superfamily, member 5, hyper-IgM syndrome) |
| 127 | CD44 | Hs.502328 | 960 | CD44 molecule (Indian blood group) |
| 128 | CD47 | Hs.446414 | 961 | CD47 molecule |
| 129 | CD69 | Hs.208854 | 969 | CD69 molecule |
| 130 | CD7 | Hs.36972 | 924 | CD7 molecule |
| 131 | CD70 | Hs.501497 | 970 | CD70 molecule |
| 132 | CD74 | Hs.436568 | 972 | CD74 molecule, major histocompatibility complex, class II invariant chain |
| 133 | CD79A | Hs.631567 | 973 | CD79a molecule, immunoglobulin-associated alpha |
| 134 | CD79B | Hs.89575 | 974 | CD79b molecule, immunoglobulin-associated beta |
| 135 | CD83 | Hs.654558 | 9308 | CD83 molecule |
| 136 | CD86 | Hs.171182 | 942 | CD86 molecule |
| 137 | CD8A | Hs.85258 | 925 | CD8a molecule |
| 138 | CD8B | Hs.405667 | 926 | CD8b molecule |
| 139 | CD93 | Hs.97199 | 22918 | CD93 molecule |
| 140 | CD96 | Hs.142023 | 10225 | CD96 molecule |
| 141 | CD97 | Hs.466039 | 976 | CD97 molecule |
| 142 | CD99 | Hs.654354 | 4267 | CD99 molecule |
| 143 | CDC42 | Hs.690198 | 998 | Cell division cycle 42 (GTP binding protein, 25kDa) |
| 144 | CDH15 | Hs.148090 | 1013 | Cadherin 15, M-cadherin (myotubule) |
| 145 | CDK6 | Hs.119882 | 1021 | Cyclin-dependent kinase 6 |
| 146 | CDKN2A | Hs.512599 | 1029 | Cyclin-dependent kinase inhibitor 2A (melanoma, p16, inhibits CDK4) |
| 147 | CEACAM1 | Hs.512682 | 634 | Carcinoembryonic antigen-related cell adhesion molecule 1 (biliary glycoprotein) |
| 148 | CEACAM8 | Hs.41 | 1088 | Carcinoembryonic antigen-related cell adhesion molecule 8 |
| 149 | CEBPA | Hs.699463 | 1050 | CCAAT/enhancer binding protein (C/EBP), alpha |
| 150 | CEBPB | Hs.517106 | 1051 | CCAAT/enhancer binding protein (C/EBP), beta |
| 151 | CEBPG | Hs.429666 | 1054 | CCAAT/enhancer binding protein (C/EBP), gamma |
| 152 | CFB | Hs.69771 | 629 | Complement factor B |
| 153 | CFD | Hs.155597 | 1675 | Complement factor D (adipsin) |
| 154 | CFHR5 | Hs.282594 | 81494 | Complement factor H-related 5 |
| 155 | CFP | Hs.53155 | 5199 | Complement factor properdin |
| 156 | CHD7 | Hs.20395 | 55636 | Chromodomain helicase DNA binding protein 7 |
| 157 | CHIA | Hs.128814 | 27159 | Chitinase, acidic |
| 158 | CHIT1 | Hs.201688 | 1118 | Chitinase 1 (chitotriosidase) |
| 159 | CHRNA4 | Hs.10734 | 1137 | Cholinergic receptor, nicotinic, alpha 4 |
| 160 | CHRNB2 | Hs.2306 | 1141 | Cholinergic receptor, nicotinic, beta 2 (neuronal) |
| 161 | CHST4 | Hs.251383 | 10164 | Carbohydrate (N-acetylglucosamine 6-O) sulfotransferase 4 |
| 162 | CHUK | Hs.198998 | 1147 | Conserved helix-loop-helix ubiquitous kinase |
| 163 | CIITA | Hs.701991 | 4261 | Class II, major histocompatibility complex, transactivator |
| 164 | CKLF | Hs.15159 | 51192 | Chemokine-like factor |
| 165 | CLEC10A | Hs.54403 | 10462 | C-type lectin domain family 10, member A |
| 166 | CLEC11A | Hs.512680 | 6320 | C-type lectin domain family 11, member A |
| 167 | CLEC16A | Hs.35490 | 23274 | C-type lectin domain family 16, member A |
| 168 | CLEC1A | Hs.29549 | 51267 | C-type lectin domain family 1, member A |
| 169 | CLEC1B | Hs.409794 | 51266 | C-type lectin domain family 1, member B |
| 170 | CLEC2B | Hs.85201 | 9976 | C-type lectin domain family 2, member B |
| 171 | CLEC2D | Hs.268326 | 29121 | C-type lectin domain family 2, member D |
| 172 | CLEC3B | Hs.476092 | 7123 | C-type lectin domain family 3, member B |
| 173 | CLEC4A | Hs.504657 | 50856 | C-type lectin domain family 4, member A |

| | | | | |
|---|---|---|---|---|
| 174 | CLEC4E | Hs.236516 | 26253 | C-type lectin domain family 4, member E |
| 175 | CLEC4M | Hs.421437 | 10332 | C-type lectin domain family 4, member M |
| 176 | CLEC5A | Hs.446235 | 23601 | C-type lectin domain family 5, member A |
| 177 | CLEC7A | Hs.143929 | 64581 | C-type lectin domain family 7, member A |
| 178 | CLPTM1 | Hs.444441 | 1209 | Cleft lip and palate associated transmembrane protein 1 |
| 179 | CMKLR1 | Hs.506659 | 1240 | Chemokine-like receptor 1 |
| 180 | CMTM6 | Hs.380627 | 54918 | CKLF-like MARVEL transmembrane domain containing 6 |
| 181 | CNIH | Hs.294603 | 10175 | Cornichon homolog (Drosophila) |
| 182 | CNR2 | Hs.73037 | 1269 | Cannabinoid receptor 2 (macrophage) |
| 183 | COL3A1 | Hs.443625 | 1281 | Collagen, type III, alpha 1 (Ehlers-Danlos syndrome type IV, autosomal dominant) |
| 184 | COL4A3BP | Hs.270437 | 10087 | Collagen, type IV, alpha 3 (Goodpasture antigen) binding protein |
| 185 | COLEC12 | Hs.464422 | 81035 | Collectin sub-family member 12 |
| 186 | CORO1A | Hs.415067 | 11151 | Coronin, actin binding protein, 1A |
| 187 | CRHR1 | Hs.417628 | 1394 | Corticotropin releasing hormone receptor 1 |
| 188 | CRISP3 | Hs.404466 | 10321 | Cysteine-rich secretory protein 3 |
| 189 | CRP | Hs.76452 | 1401 | C-reactive protein, pentraxin-related |
| 190 | CRTAM | Hs.159523 | 56253 | Cytotoxic and regulatory T cell molecule |
| 191 | CSF1 | Hs.591402 | 1435 | Colony stimulating factor 1 (macrophage) |
| 192 | CSF1R | Hs.654394 | 1436 | Colony stimulating factor 1 receptor, formerly McDonough feline sarcoma viral (v-fms) oncogene hom |
| 193 | CSF2 | Hs.1349 | 1437 | Colony stimulating factor 2 (granulocyte-macrophage) |
| 194 | CSF2RA | Hs.520937 | 1438 | Colony stimulating factor 2 receptor, alpha, low-affinity (granulocyte-macrophage) |
| 195 | CSF2RB | Hs.592192 | 1439 | Colony stimulating factor 2 receptor, beta, low-affinity (granulocyte-macrophage) |
| 196 | CSF3 | Hs.2233 | 1440 | Colony stimulating factor 3 (granulocyte) |
| 197 | CSF3R | Hs.524517 | 1441 | Colony stimulating factor 3 receptor (granulocyte) |
| 198 | CST7 | Hs.143212 | 8530 | Cystatin F (leukocystatin) |
| 199 | CTLA4 | Hs.247824 | 1493 | Cytotoxic T-lymphocyte-associated protein 4 |
| 200 | CTSC | Hs.128065 | 1075 | Cathepsin C |
| 201 | CTSE | Hs.701979 | 1510 | Cathepsin E |
| 202 | CTSG | Hs.421724 | 1511 | Cathepsin G |
| 203 | CTSS | Hs.181301 | 1520 | Cathepsin S |
| 204 | CTSW | Hs.416848 | 1521 | Cathepsin W |
| 205 | CX3CL1 | Hs.531668 | 6376 | Chemokine (C-X3-C motif) ligand 1 |
| 206 | CX3CR1 | Hs.78913 | 1524 | Chemokine (C-X3-C motif) receptor 1 |
| 207 | CXADR | Hs.705503 | 1525 | Coxsackie virus and adenovirus receptor |
| 208 | CXCL10 | Hs.632586 | 3627 | Chemokine (C-X-C motif) ligand 10 |
| 209 | CXCL11 | Hs.632592 | 6373 | Chemokine (C-X-C motif) ligand 11 |
| 210 | CXCL12 | Hs.522891 | 6387 | Chemokine (C-X-C motif) ligand 12 (stromal cell-derived factor 1) |
| 211 | CXCL13 | Hs.100431 | 10563 | Chemokine (C-X-C motif) ligand 13 (B-cell chemoattractant) |
| 212 | CXCL14 | Hs.483444 | 9547 | Chemokine (C-X-C motif) ligand 14 |
| 213 | CXCL2 | Hs.590921 | 2920 | Chemokine (C-X-C motif) ligand 2 |
| 214 | CXCL3 | Hs.89690 | 2921 | Chemokine (C-X-C motif) ligand 3 |
| 215 | CXCL5 | Hs.89714 | 6374 | Chemokine (C-X-C motif) ligand 5 |
| 216 | CXCL6 | Hs.164021 | 6372 | Chemokine (C-X-C motif) ligand 6 (granulocyte chemotactic protein 2) |
| 217 | CXCL9 | Hs.77367 | 4283 | Chemokine (C-X-C motif) ligand 9 |
| 218 | CXCR1 | Hs.194778 | 3577 | Chemokine (C-X-C motif) receptor 1 |
| 219 | CXCR2 | Hs.846 | 3579 | Chemokine (C-X-C motif) receptor 2 |
| 220 | CXCR3 | Hs.198252 | 2833 | Chemokine (C-X-C motif) receptor 3 |
| 221 | CXCR4 | Hs.593413 | 7852 | Chemokine (C-X-C motif) receptor 4 |
| 222 | CXCR5 | Hs.113916 | 643 | Chemokine (C-X-C motif) receptor 5 |
| 223 | CXCR6 | Hs.34526 | 10663 | Chemokine (C-X-C motif) receptor 6 |
| 224 | CXCR7 | Hs.471751 | 57007 | Chemokine (C-X-C motif) receptor 7 |
| 225 | CYBA | Hs.513803 | 1535 | Cytochrome b-245, alpha polypeptide |
| 226 | CYBB | Hs.292356 | 1536 | Cytochrome b-245, beta polypeptide (chronic granulomatous disease) |
| 227 | CYP11B1 | Hs.184927 | 1584 | Cytochrome P450, family 11, subfamily B, polypeptide 1 |
| 228 | CYSLTR2 | Hs.253706 | 57105 | Cysteinyl leukotriene receptor 2 |
| 229 | DCAF15 | Hs.443636 | 90379 | DDB1- and CUL4-associated factor 15 |
| 230 | DDOST | Hs.523145 | 1650 | Dolichyl-diphosphooligosaccharide-protein glycosyltransferase |
| 231 | DEFB4A | Hs.740237 | 100289462 | Defensin beta 4A |

| | | | | |
|---|---|---|---|---|
| 232 | DHRS2 | Hs.272499 | 10202 | Dehydrogenase/reductase (SDR family) member 2 |
| 233 | DMBT1 | Hs.279611 | 1755 | Deleted in malignant brain tumors 1 |
| 234 | DOCK2 | Hs.586174 | 1794 | Dedicator of cytokinesis 2 |
| 235 | DPP4 | Hs.368912 | 1803 | Dipeptidyl-peptidase 4 (CD26, adenosine deaminase complexing protein 2) |
| 236 | DPP8 | Hs.591106 | 54878 | Dipeptidyl-peptidase 8 |
| 237 | DYRK3 | Hs.164267 | 8444 | Dual-specificity tyrosine-(Y)-phosphorylation regulated kinase 3 |
| 238 | EBI3 | Hs.501452 | 10148 | Epstein-Barr virus induced gene 3 |
| 239 | EDA | Hs.105407 | 1896 | Ectodysplasin A |
| 240 | EDA2R | Hs.302017 | 60401 | Ectodysplasin A2 receptor |
| 241 | EDAR | Hs.171971 | 10913 | Ectodysplasin A receptor |
| 242 | EDN1 | Hs.511899 | 1906 | Endothelin 1 |
| 243 | EDN2 | Hs.1407 | 1907 | Endothelin 2 |
| 244 | EDN3 | Hs.1408 | 1908 | Endothelin 3 |
| 245 | EDNRB | Hs.82002 | 1910 | Endothelin receptor type B |
| 246 | EGF | Hs.419815 | 1950 | Epidermal growth factor (beta-urogastrone) |
| 247 | EGFR | Hs.488293 | 1956 | Epidermal growth factor receptor (erythroblastic leukemia viral (v-erb-b) oncogene homolog, avian) |
| 248 | ELF4 | Hs.271940 | 2000 | E74-like factor 4 (ets domain transcription factor) |
| 249 | EPHB6 | Hs.380089 | 2051 | EPH receptor B6 |
| 250 | ERAP1 | Hs.436186 | 51752 | Endoplasmic reticulum aminopeptidase 1 |
| 251 | ERAP2 | Hs.591249 | 64167 | Endoplasmic reticulum aminopeptidase 2 |
| 252 | ERBB2 | Hs.446352 | 2064 | V-erb-b2 erythroblastic leukemia viral oncogene homolog 2, neuro/glioblastoma derived oncogene ho |
| 253 | ERBB3 | Hs.118681 | 2065 | V-erb-b2 erythroblastic leukemia viral oncogene homolog 3 (avian) |
| 254 | ERBB4 | Hs.390729 | 2066 | V-erb-a erythroblastic leukemia viral oncogene homolog 4 (avian) |
| 255 | EREG | Hs.115263 | 2069 | Epiregulin |
| 256 | ETS1 | Hs.369438 | 2113 | V-ets erythroblastosis virus E26 oncogene homolog 1 (avian) |
| 257 | EXOSC9 | Hs.91728 | 5393 | Exosome component 9 |
| 258 | F12 | Hs.1321 | 2161 | Coagulation factor XII (Hageman factor) |
| 259 | FAS | Hs.244139 | 355 | Fas (TNF receptor superfamily, member 6) |
| 260 | FASLG | Hs.2007 | 356 | Fas ligand (TNF superfamily, member 6) |
| 261 | FCAR | Hs.659872 | 2204 | Fc fragment of IgA, receptor for |
| 262 | FCGR1B | Hs.534956 | 2210 | Fc fragment of IgG, high affinity Ib, receptor (CD64) |
| 263 | FCGR2A | Hs.352642 | 2212 | Fc fragment of IgG, low affinity IIa, receptor (CD32) |
| 264 | FCGR2B | Hs.654395 | 2213 | Fc fragment of IgG, low affinity IIb, receptor (CD32) |
| 265 | FCGR2C | Hs.705497 | 9103 | Fc fragment of IgG, low affinity IIc, receptor for (CD32) |
| 266 | FCGR3B | Hs.694258 | 2215 | Fc fragment of IgG, low affinity IIIb, receptor for (CD16) |
| 267 | FCGRT | Hs.111903 | 2217 | Fc fragment of IgG, receptor, transporter, alpha |
| 268 | FCN1 | Hs.440898 | 2219 | Ficolin (collagen/fibrinogen domain containing) 1 |
| 269 | FCN2 | Hs.54517 | 2220 | Ficolin (collagen/fibrinogen domain containing lectin) 2 (hucolin) |
| 270 | FGF1 | Hs.483635 | 2246 | Fibroblast growth factor 1 (acidic) |
| 271 | FGF12 | Hs.584758 | 2257 | Fibroblast growth factor 12 |
| 272 | FGF13 | Hs.6540 | 2258 | Fibroblast growth factor 13 |
| 273 | FGF14 | Hs.696392 | 2259 | Fibroblast growth factor 14 |
| 274 | FGF16 | Hs.666364 | 8823 | Fibroblast growth factor 16 |
| 275 | FGF17 | Hs.248192 | 8822 | Fibroblast growth factor 17 |
| 276 | FGF18 | Hs.87191 | 8817 | Fibroblast growth factor 18 |
| 277 | FGF2 | Hs.284244 | 2247 | Fibroblast growth factor 2 (basic) |
| 278 | FGF20 | Hs.199905 | 26281 | Fibroblast growth factor 20 |
| 279 | FGF21 | Hs.283015 | 26291 | Fibroblast growth factor 21 |
| 280 | FGF22 | Hs.248087 | 27006 | Fibroblast growth factor 22 |
| 281 | FGF23 | Hs.287370 | 8074 | Fibroblast growth factor 23 |
| 282 | FGF3 | Hs.37092 | 2248 | Fibroblast growth factor 3 (murine mammary tumor virus integration site (v-int-2) oncogene homolog) |
| 283 | FGF5 | Hs.37055 | 2250 | Fibroblast growth factor 5 |
| 284 | FGF6 | Hs.166015 | 2251 | Fibroblast growth factor 6 |
| 285 | FGF7 | Hs.567268 | 2252 | Fibroblast growth factor 7 (keratinocyte growth factor) |
| 286 | FGF8 | Hs.57710 | 2253 | Fibroblast growth factor 8 (androgen-induced) |
| 287 | FGF9 | Hs.111 | 2254 | Fibroblast growth factor 9 (glia-activating factor) |
| 288 | FGFR1 | Hs.264887 | 2260 | Fibroblast growth factor receptor 1 (fms-related tyrosine kinase 2, Pfeiffer syndrome) |
| 289 | FGFR2 | Hs.533683 | 2263 | Fibroblast growth factor receptor 2 (bacteria-expressed kinase, keratinocyte growth factor receptor, c syndrome) |

| | | | | |
|---|---|---|---|---|
| 290 | FGFR3 | Hs.1420 | 2261 | Fibroblast growth factor receptor 3 (achondroplasia, thanatophoric dwarfism) |
| 291 | FGFR4 | Hs.165950 | 2264 | Fibroblast growth factor receptor 4 |
| 292 | FIGF | Hs.11392 | 2277 | C-fos induced growth factor (vascular endothelial growth factor D) |
| 293 | FKBP1A | Hs.471933 | 2280 | FK506 binding protein 1A, 12kDa |
| 294 | FLT3 | Hs.507590 | 2322 | Fms-related tyrosine kinase 3 |
| 295 | FLT4 | Hs.646917 | 2324 | Fms-related tyrosine kinase 4 |
| 296 | FOXO3 | Hs.220950 | 2309 | Forkhead box O3 |
| 297 | FOXP3 | Hs.247700 | 50943 | Forkhead box P3 |
| 298 | FTH1 | Hs.524910 | 2495 | Ferritin, heavy polypeptide 1 |
| 299 | FUT4 | Hs.390420 | 2526 | Fucosyltransferase 4 (alpha (1,3) fucosyltransferase, myeloid-specific) |
| 300 | FUT9 | Hs.49117 | 10690 | Fucosyltransferase 9 (alpha (1,3) fucosyltransferase) |
| 301 | FYB | Hs.370503 | 2533 | FYN binding protein (FYB-120/130) |
| 302 | FYN | Hs.390567 | 2534 | FYN oncogene related to SRC, FGR, YES |
| 303 | G6PD | Hs.461047 | 2539 | Glucose-6-phosphate dehydrogenase |
| 304 | GALNT2 | Hs.654649 | 2590 | UDP-N-acetyl-alpha-D-galactosamine:polypeptide N-acetylgalactosaminyltransferase 2 (GalNAc-T2) |
| 305 | GBP2 | Hs.386567 | 2634 | Guanylate binding protein 2, interferon-inducible |
| 306 | GCH1 | Hs.86724 | 2643 | GTP cyclohydrolase 1 (dopa-responsive dystonia) |
| 307 | GEM | Hs.654463 | 2669 | GTP binding protein overexpressed in skeletal muscle |
| 308 | GFRA2 | Hs.441202 | 2675 | GDNF family receptor alpha 2 |
| 309 | GLMN | Hs.49105 | 11146 | Glomulin, FKBP associated protein |
| 310 | GNL1 | Hs.83147 | 2794 | Guanine nucleotide binding protein-like 1 |
| 311 | GNLY | Hs.105806 | 10578 | Granulysin |
| 312 | GPI | Hs.466471 | 2821 | Glucose phosphate isomerase |
| 313 | GPR183 | Hs.784 | 1880 | G-protein coupled receptor 183 |
| 314 | GPR44 | Hs.299567 | 11251 | G protein-coupled receptor 44 |
| 315 | GPR65 | Hs.513440 | 8477 | G protein-coupled receptor 65 |
| 316 | GTPBP1 | Hs.276925 | 9567 | GTP binding protein 1 |
| 317 | GZMA | Hs.90708 | 3001 | Granzyme A (granzyme 1, cytotoxic T-lymphocyte-associated serine esterase 3) |
| 318 | GZMB | Hs.1051 | 3002 | Granzyme B (granzyme 2, cytotoxic T-lymphocyte-associated serine esterase 1) |
| 319 | GZMH | Hs.348264 | 2999 | Granzyme H (cathepsin G-like 2, protein h-CCPX) |
| 320 | GZMK | Hs.277937 | 3003 | Granzyme K (granzyme 3; tryptase II) |
| 321 | GZMM | Hs.465511 | 3004 | Granzyme M (lymphocyte met-ase 1) |
| 322 | HAMP | Hs.8821 | 57817 | Hepcidin antimicrobial peptide |
| 323 | HCLS1 | Hs.14601 | 3059 | Hematopoietic cell-specific Lyn substrate 1 |
| 324 | HDAC4 | Hs.20516 | 9759 | Histone deacetylase 4 |
| 325 | HDAC5 | Hs.438782 | 10014 | Histone deacetylase 5 |
| 326 | HDAC7 | Hs.200063 | 51564 | Histone deacetylase 7 |
| 327 | HDAC9 | Hs.196054 | 9734 | Histone deacetylase 9 |
| 328 | HELLS | Hs.655830 | 3070 | Helicase, lymphoid-specific |
| 329 | HGF | Hs.396530 | 3082 | Hepatocyte growth factor (hepapoietin A; scatter factor) |
| 330 | HIF1A | Hs.597216 | 3091 | Hypoxia-inducible factor 1, alpha subunit (basic helix-loop-helix transcription factor) |
| 331 | HIF1AN | Hs.500788 | 55662 | Hypoxia-inducible factor 1, alpha subunit inhibitor |
| 332 | HIF3A | Hs.420830 | 64344 | Hypoxia inducible factor 3, alpha subunit |
| 333 | HLA-A | Hs.181244 | 3105 | Major histocompatibility complex, class I, A |
| 334 | HLA-B | Hs.77961 | 3106 | Major histocompatibility complex, class I, B |
| 335 | HLA-C | Hs.449621 | 3107 | Major histocompatibility complex, class I, C |
| 336 | HLA-DPA1 | Hs.347270 | 3113 | Major histocompatibility complex, class II, DP alpha 1 |
| 337 | HLA-DQA1 | Hs.387679 | 3117 | Major histocompatibility complex, class II, DQ alpha 1 |
| 338 | HLA-DQB1 | Hs.409934 | 3119 | Major histocompatibility complex, class II, DQ beta 1 |
| 339 | HLA-DQB2 | Hs.731563 | 3120 | Major histocompatibility complex, class II, DQ beta 2 |
| 340 | HLA-DRA | Hs.520048 | 3122 | Major histocompatibility complex, class II, DR alpha |
| 341 | HLA-E | Hs.650174 | 3133 | Major histocompatibility complex, class I, E |
| 342 | HLA-F | Hs.519972 | 3134 | Major histocompatibility complex, class I, F |
| 343 | HLA-G | Hs.512152 | 3135 | HLA-G histocompatibility antigen, class I, G |
| 344 | HMGB1 | Hs.434102 | 3146 | High-mobility group box 1 |
| 345 | HMOX1 | Hs.517581 | 3162 | Heme oxygenase (decycling) 1 |
| 346 | HRH2 | Hs.247885 | 3274 | Histamine receptor H2 |
| 347 | HSP90AA1 | Hs.523560 | 3320 | Heat shock protein 90kDa alpha (cytosolic), class A member 1 |

| | | | | |
|---|---|---|---|---|
| 348 | HSP90AB1 | Hs.509736 | 3326 | Heat shock protein 90kDa alpha (cytosolic), class B member 1 |
| 349 | HSPA1A | Hs.520028 | 3303 | Heat shock 70kDa protein 1A |
| 350 | HSPD1 | Hs.595053 | 3329 | Heat shock 60kDa protein 1 (chaperonin) |
| 351 | ICAM1 | Hs.643447 | 3383 | Intercellular adhesion molecule 1 (CD54), human rhinovirus receptor |
| 352 | ICOS | Hs.56247 | 29851 | Inducible T-cell co-stimulator |
| 353 | ICOSLG | Hs.14155 | 23308 | Inducible T-cell co-stimulator ligand |
| 354 | IDO1 | Hs.840 | 3620 | Indoleamine 2,3-dioxygenase 1 |
| 355 | IFI16 | Hs.380250 | 3428 | Interferon, gamma-inducible protein 16 |
| 356 | IFI35 | Hs.632258 | 3430 | Interferon-induced protein 35 |
| 357 | IFI6 | Hs.511731 | 2537 | Interferon, alpha-inducible protein 6 |
| 358 | IFITM2 | Hs.458414 | 10581 | Interferon induced transmembrane protein 2 (1-8D) |
| 359 | IFITM3 | Hs.374650 | 10410 | Interferon induced transmembrane protein 3 (1-8U) |
| 360 | IFNA1 | Hs.37026 | 3439 | Interferon, alpha 1 |
| 361 | IFNA10 | Hs.282275 | 3446 | Interferon, alpha 10 |
| 362 | IFNA14 | Hs.93907 | 3448 | Interferon, alpha 14 |
| 363 | IFNA16 | Hs.56303 | 3449 | Interferon, alpha 16 |
| 364 | IFNA17 | Hs.282276 | 3451 | Interferon, alpha 17 |
| 365 | IFNA2 | Hs.211575 | 3440 | Interferon, alpha 2 |
| 366 | IFNA21 | Hs.113211 | 3452 | Interferon, alpha 21 |
| 367 | IFNA4 | Hs.1510 | 3441 | Interferon, alpha 4 |
| 368 | IFNA5 | Hs.37113 | 3442 | Interferon, alpha 5 |
| 369 | IFNA6 | Hs.533470 | 3443 | Interferon, alpha 6 |
| 370 | IFNA7 | Hs.282274 | 3444 | Interferon, alpha 7 |
| 371 | IFNA8 | Hs.73890 | 3445 | Interferon, alpha 8 |
| 372 | IFNAR1 | Hs.529400 | 3454 | Interferon (alpha, beta and omega) receptor 1 |
| 373 | IFNAR2 | Hs.701988 | 3455 | Interferon (alpha, beta and omega) receptor 2 |
| 374 | IFNB1 | Hs.93177 | 3456 | Interferon, beta 1, fibroblast |
| 375 | IFNG | Hs.856 | 3458 | Interferon, gamma |
| 376 | IFNGR1 | Hs.520414 | 3459 | Interferon gamma receptor 1 |
| 377 | IFNGR2 | Hs.634632 | 3460 | Interferon gamma receptor 2 (interferon gamma transducer 1) |
| 378 | IFNW1 | Hs.73010 | 3467 | Interferon, omega 1 |
| 379 | IGF1R | Hs.643120 | 3480 | Insulin-like growth factor 1 receptor |
| 380 | IGF2R | Hs.487062 | 3482 | Insulin-like growth factor 2 receptor |
| 381 | IGFBP3 | Hs.450230 | 3486 | Insulin-like growth factor binding protein 3 |
| 382 | IGHA1 | Hs.648398 | 3493 | Immunoglobulin heavy constant alpha 1 |
| 383 | IGHD | Hs.510635 | 3495 | Immunoglobulin heavy constant delta |
| 384 | IGHM | - | 3507 | Immunoglobulin heavy constant mu |
| 385 | IGJ | Hs.700610 | 3512 | Immunoglobulin J polypeptide, linker protein for immunoglobulin alpha and mu polypeptides |
| 386 | IGKV4-1 | - | 28908 | Immunoglobulin kappa variable 4-1 |
| 387 | IGLL1 | Hs.348935 | 3543 | Immunoglobulin lambda-like polypeptide 1 |
| 388 | IGSF6 | Hs.530902 | 10261 | Immunoglobulin superfamily, member 6 |
| 389 | IK | Hs.421245 | 3550 | IK cytokine, down-regulator of HLA II |
| 390 | IKBKAP | Hs.494738 | 8518 | Inhibitor of kappa light polypeptide gene enhancer in B-cells, kinase complex-associated protein |
| 391 | IKBKE | Hs.321045 | 9641 | Inhibitor of kappa light polypeptide gene enhancer in B-cells, kinase epsilon |
| 392 | IKBKG | Hs.43505 | 8517 | Inhibitor of kappa light polypeptide gene enhancer in B-cells, kinase gamma |
| 393 | IL10 | Hs.193717 | 3586 | Interleukin 10 |
| 394 | IL10RA | Hs.504035 | 3587 | Interleukin 10 receptor, alpha |
| 395 | IL10RB | Hs.654593 | 3588 | Interleukin 10 receptor, beta |
| 396 | IL11 | Hs.467304 | 3589 | Interleukin 11 |
| 397 | IL11RA | Hs.591088 | 3590 | Interleukin 11 receptor, alpha |
| 398 | IL12A | Hs.673 | 3592 | Interleukin 12A (natural killer cell stimulatory factor 1, cytotoxic lymphocyte maturation factor 1, p35) |
| 399 | IL12B | Hs.674 | 3593 | Interleukin 12B (natural killer cell stimulatory factor 2, cytotoxic lymphocyte maturation factor 2, p40) |
| 400 | IL12RB1 | Hs.567294 | 3594 | Interleukin 12 receptor, beta 1 |
| 401 | IL12RB2 | Hs.479347 | 3595 | Interleukin 12 receptor, beta 2 |
| 402 | IL13 | Hs.845 | 3596 | Interleukin 13 |
| 403 | IL13RA1 | Hs.496646 | 3597 | Interleukin 13 receptor, alpha 1 |
| 404 | IL13RA2 | Hs.336046 | 3598 | Interleukin 13 receptor, alpha 2 |
| 405 | IL15 | Hs.654378 | 3600 | Interleukin 15 |

| | | | | |
|---|---|---|---|---|
| 406 | IL15RA | Hs.524117 | 3601 | Interleukin 15 receptor, alpha |
| 407 | IL16 | Hs.459095 | 3603 | Interleukin 16 (lymphocyte chemoattractant factor) |
| 408 | IL17A | Hs.41724 | 3605 | Interleukin 17A |
| 409 | IL17B | Hs.156979 | 27190 | Interleukin 17B |
| 410 | IL17RA | Hs.129751 | 23765 | Interleukin 17 receptor A |
| 411 | IL17RB | Hs.654970 | 55540 | Interleukin 17 receptor B |
| 412 | IL17RC | Hs.129959 | 84818 | Interleukin 17 receptor C |
| 413 | IL18 | Hs.83077 | 3606 | Interleukin 18 (interferon-gamma-inducing factor) |
| 414 | IL18R1 | Hs.469521 | 8809 | Interleukin 18 receptor 1 |
| 415 | IL18RAP | Hs.158315 | 8807 | Interleukin 18 receptor accessory protein |
| 416 | IL19 | Hs.661017 | 29949 | Interleukin 19 |
| 417 | IL1A | Hs.1722 | 3552 | Interleukin 1, alpha |
| 418 | IL1B | Hs.126256 | 3553 | Interleukin 1, beta |
| 419 | IL1F6 | Hs.278910 | 27179 | Interleukin 1 family, member 6 (epsilon) |
| 420 | IL1F7 | Hs.166371 | 27178 | Interleukin 1 family, member 7 (zeta) |
| 421 | IL1R1 | Hs.701982 | 3554 | Interleukin 1 receptor, type I |
| 422 | IL1R2 | Hs.25333 | 7850 | Interleukin 1 receptor, type II |
| 423 | IL1RAP | Hs.478673 | 3556 | Interleukin 1 receptor accessory protein |
| 424 | IL1RAPL1 | Hs.658912 | 11141 | Interleukin 1 receptor accessory protein-like 1 |
| 425 | IL1RAPL2 | Hs.675519 | 26280 | Interleukin 1 receptor accessory protein-like 2 |
| 426 | IL1RL1 | Hs.66 | 9173 | Interleukin 1 receptor-like 1 |
| 427 | IL1RL2 | Hs.659863 | 8808 | Interleukin 1 receptor-like 2 |
| 428 | IL1RN | Hs.81134 | 3557 | Interleukin 1 receptor antagonist |
| 429 | IL2 | Hs.89679 | 3558 | Interleukin 2 |
| 430 | IL20RA | Hs.445868 | 53832 | Interleukin 20 receptor, alpha |
| 431 | IL21 | Hs.567559 | 59067 | Interleukin 21 |
| 432 | IL21R | Hs.210546 | 50615 | Interleukin 21 receptor |
| 433 | IL22 | Hs.287369 | 50616 | Interleukin 22 |
| 434 | IL22RA1 | Hs.110915 | 58985 | Interleukin 22 receptor, alpha 1 |
| 435 | IL23A | Hs.98309 | 51561 | Interleukin 23, alpha subunit p19 |
| 436 | IL24 | Hs.58831 | 11009 | Interleukin 24 |
| 437 | IL25 | Hs.302036 | 64806 | Interleukin 25 |
| 438 | IL26 | Hs.272350 | 55801 | Interleukin 26 |
| 439 | IL27RA | Hs.132781 | 9466 | Interleukin 27 receptor, alpha |
| 440 | IL2RA | Hs.231367 | 3559 | Interleukin 2 receptor, alpha |
| 441 | IL2RB | Hs.474787 | 3560 | Interleukin 2 receptor, beta |
| 442 | IL2RG | Hs.84 | 3561 | Interleukin 2 receptor, gamma (severe combined immunodeficiency) |
| 443 | IL3 | Hs.694 | 3562 | Interleukin 3 (colony-stimulating factor, multiple) |
| 444 | IL32 | Hs.943 | 9235 | Interleukin 32 |
| 445 | IL33 | Hs.348390 | 90865 | Interleukin 33 |
| 446 | IL3RA | Hs.632790 | 3563 | Interleukin 3 receptor, alpha (low affinity) |
| 447 | IL4 | Hs.73917 | 3565 | Interleukin 4 |
| 448 | IL4R | Hs.513457 | 3566 | Interleukin 4 receptor |
| 449 | IL5 | Hs.2247 | 3567 | Interleukin 5 (colony-stimulating factor, eosinophil) |
| 450 | IL5RA | Hs.68876 | 3568 | Interleukin 5 receptor, alpha |
| 451 | IL6 | Hs.654458 | 3569 | Interleukin 6 (interferon, beta 2) |
| 452 | IL6R | Hs.695954 | 3570 | Interleukin 6 receptor |
| 453 | IL6ST | Hs.532082 | 3572 | Interleukin 6 signal transducer (gp130, oncostatin M receptor) |
| 454 | IL7 | Hs.591873 | 3574 | Interleukin 7 |
| 455 | IL7R | Hs.591742 | 3575 | Interleukin 7 receptor |
| 456 | IL8 | Hs.624 | 3576 | Interleukin 8 |
| 457 | IL9 | Hs.960 | 3578 | Interleukin 9 |
| 458 | INHA | Hs.407506 | 3623 | Inhibin, alpha |
| 459 | INHBA | Hs.583348 | 3624 | Inhibin, beta A |
| 460 | INS | Hs.654579 | 3630 | Insulin |
| 461 | IRF1 | Hs.436061 | 3659 | Interferon regulatory factor 1 |
| 462 | IRF4 | Hs.401013 | 3662 | Interferon regulatory factor 4 |
| 463 | IRF8 | Hs.137427 | 3394 | Interferon regulatory factor 8 |

| | | | | |
|---|---|---|---|---|
| 464 | ITGA4 | Hs.694732 | 3676 | Integrin, alpha 4 (antigen CD49D, alpha 4 subunit of VLA-4 receptor) |
| 465 | ITGAE | Hs.513867 | 3682 | Integrin, alpha E (antigen CD103, human mucosal lymphocyte antigen 1; alpha polypeptide) |
| 466 | ITGAL | Hs.174103 | 3683 | Integrin, alpha L (antigen CD11A (p180), lymphocyte function-associated antigen 1; alpha polypeptid |
| 467 | ITGAM | Hs.172631 | 3684 | Integrin, alpha M (complement component 3 receptor 3 subunit) |
| 468 | ITGAX | Hs.248472 | 3687 | Integrin, alpha X (complement component 3 receptor 4 subunit) |
| 469 | ITGB1 | Hs.695946 | 3688 | Integrin, beta 1 (fibronectin receptor, beta polypeptide, antigen CD29 includes MDF2, MSK12) |
| 470 | ITGB2 | Hs.375957 | 3689 | Integrin, beta 2 (complement component 3 receptor 3 and 4 subunit) |
| 471 | ITGB7 | Hs.654470 | 3695 | Integrin, beta 7 |
| 472 | ITIH1 | Hs.420257 | 3697 | Inter-alpha (globulin) inhibitor H1 |
| 473 | JAG1 | Hs.224012 | 182 | Jagged 1 (Alagille syndrome) |
| 474 | JAG2 | Hs.433445 | 3714 | Jagged 2 |
| 475 | KDR | Hs.479756 | 3791 | Kinase insert domain receptor (a type III receptor tyrosine kinase) |
| 476 | KIF13B | Hs.444767 | 23303 | Kinesin family member 13B |
| 477 | KIR2DL1 | Hs.654605 | 3802 | Killer cell immunoglobulin-like receptor, two domains, long cytoplasmic tail, 1 |
| 478 | KIR2DL2 | - | 3803 | Killer cell immunoglobulin-like receptor, two domains, long cytoplasmic tail, 2 |
| 479 | KIR2DL3 | Hs.731378 | 3804 | Killer cell immunoglobulin-like receptor, two domains, long cytoplasmic tail, 3 |
| 480 | KIR2DL4 | Hs.651287 | 3805 | Killer cell immunoglobulin-like receptor, two domains, long cytoplasmic tail, 4 |
| 481 | KIR2DL5A | Hs.676464 | 57292 | Killer cell immunoglobulin-like receptor, two domains, long cytoplasmic tail, 5A |
| 482 | KIR2DS1 | Hs.720721 | 3806 | Killer cell immunoglobulin-like receptor, two domains, short cytoplasmic tail, 1 |
| 483 | KIR2DS3 | - | 3808 | Killer cell immunoglobulin-like receptor, two domains, short cytoplasmic tail, 3 |
| 484 | KIR2DS4 | Hs.654608 | 3809 | Killer cell immunoglobulin-like receptor, two domains, short cytoplasmic tail, 4 |
| 485 | KIR3DL1 | Hs.645228 | 3811 | Killer cell immunoglobulin-like receptor, three domains, long cytoplasmic tail, 1 |
| 486 | KIR3DL3 | Hs.645224 | 115653 | Killer cell immunoglobulin-like receptor, three domains, long cytoplasmic tail, 3 |
| 487 | KIR3DS1 | Hs.659860 | 3813 | Killer cell immunoglobulin-like receptor, three domains, short cytoplasmic tail, 1 |
| 488 | KIT | Hs.479754 | 3815 | V-kit Hardy-Zuckerman 4 feline sarcoma viral oncogene homolog |
| 489 | KITLG | Hs.1048 | 4254 | KIT ligand |
| 490 | KLF6 | Hs.4055 | 1316 | Kruppel-like factor 6 |
| 491 | KLRB1 | Hs.169824 | 3820 | Killer cell lectin-like receptor subfamily B, member 1 |
| 492 | KLRC3 | Hs.654362 | 3823 | Killer cell lectin-like receptor subfamily C, member 3 |
| 493 | KLRC4 | Hs.665704 | 8302 | Killer cell lectin-like receptor subfamily C, member 4 |
| 494 | KLRD1 | Hs.562457 | 3824 | Killer cell lectin-like receptor subfamily D, member 1 |
| 495 | KLRF1 | Hs.183125 | 51348 | Killer cell lectin-like receptor subfamily F, member 1 |
| 496 | KLRG1 | Hs.558446 | 10219 | Killer cell lectin-like receptor subfamily G, member 1 |
| 497 | KLRK1 | Hs.387787 | 22914 | Killer cell lectin-like receptor subfamily K, member 1 |
| 498 | KRT1 | Hs.80828 | 3848 | Keratin 1 (epidermolytic hyperkeratosis) |
| 499 | KYNU | Hs.470126 | 8942 | Kynureninase (L-kynurenine hydrolase) |
| 500 | LAG3 | Hs.409523 | 3902 | Lymphocyte-activation gene 3 |
| 501 | LAIR1 | Hs.572535 | 3903 | Leukocyte-associated immunoglobulin-like receptor 1 |
| 502 | LAIR2 | Hs.43803 | 3904 | Leukocyte-associated immunoglobulin-like receptor 2 |
| 503 | LAMP3 | Hs.518448 | 27074 | Lysosomal-associated membrane protein 3 |
| 504 | LAT2 | Hs.647049 | 7462 | Linker for activation of T cells family, member 2 |
| 505 | LAX1 | Hs.272794 | 54900 | Lymphocyte transmembrane adaptor 1 |
| 506 | LBP | Hs.154078 | 3929 | Lipopolysaccharide binding protein |
| 507 | LCK | Hs.470627 | 3932 | Lymphocyte-specific protein tyrosine kinase |
| 508 | LCP2 | Hs.304475 | 3937 | Lymphocyte cytosolic protein 2 (SH2 domain containing leukocyte protein of 76kDa) |
| 509 | LDB1 | Hs.702266 | 8861 | LIM domain binding 1 |
| 510 | LIF | Hs.2250 | 3976 | Leukemia inhibitory factor (cholinergic differentiation factor) |
| 511 | LIFR | Hs.133421 | 3977 | Leukemia inhibitory factor receptor alpha |
| 512 | LIG1 | Hs.1770 | 3978 | Ligase I, DNA, ATP-dependent |
| 513 | LIG3 | Hs.100299 | 3980 | Ligase III, DNA, ATP-dependent |
| 514 | LIG4 | Hs.166091 | 3981 | Ligase IV, DNA, ATP-dependent |
| 515 | LILRA1 | Hs.710507 | 11024 | Leukocyte immunoglobulin-like receptor, subfamily A (without TM domain), member 1 |
| 516 | LILRA2 | Hs.655593 | 11027 | Leukocyte immunoglobulin-like receptor, subfamily A (without TM domain), member 2 |
| 517 | LILRA3 | Hs.113277 | 11026 | Leukocyte immunoglobulin-like receptor, subfamily A (without TM domain), member 3 |
| 518 | LILRA4 | Hs.406708 | 23547 | Leukocyte immunoglobulin-like receptor, subfamily A (without TM domain), member 4 |
| 519 | LILRA5 | Hs.710986 | 353514 | Leukocyte immunoglobulin-like receptor, subfamily A (without TM domain), member 5 |
| 520 | LILRA6 | Hs.688335 | 79168 | Leukocyte immunoglobulin-like receptor, subfamily A (without TM domain), member 6 |
| 521 | LILRB1 | Hs.655593 | 10859 | Leukocyte immunoglobulin-like receptor, subfamily B (with TM and ITIM domains), member 1 |

| | | | | |
|---|---|---|---|---|
| 522 | LILRB2 | Hs.655652 | 10288 | Leukocyte immunoglobulin-like receptor, subfamily B (with TM and ITIM domains), member 2 |
| 523 | LILRB3 | Hs.631592 | 11025 | Leukocyte immunoglobulin-like receptor, subfamily B (with TM and ITIM domains), member 3 |
| 524 | LILRB4 | Hs.67846 | 11006 | Leukocyte immunoglobulin-like receptor, subfamily B (with TM and ITIM domains), member 4 |
| 525 | LILRB5 | Hs.655338 | 10990 | Leukocyte immunoglobulin-like receptor, subfamily B (with TM and ITIM domains), member 5 |
| 526 | LOC254896 | Hs.735240 | 254896 | Uncharacterized LOC254896 |
| 527 | LOC91316 | Hs.148656 | 91316 | Similar to bK246H3.1 (immunoglobulin lambda-like polypeptide 1, pre-B-cell specific) |
| 528 | LTA | Hs.36 | 4049 | Lymphotoxin alpha (TNF superfamily, member 1) |
| 529 | LTB | Hs.376208 | 4050 | Lymphotoxin beta (TNF superfamily, member 3) |
| 530 | LTB4R | Hs.655431 | 1241 | Leukotriene B4 receptor |
| 531 | LTB4R2 | Hs.642693 | 56413 | Leukotriene B4 receptor 2 |
| 532 | LTBR | Hs.1116 | 4055 | Lymphotoxin beta receptor (TNFR superfamily, member 3) |
| 533 | LTF | Hs.529517 | 4057 | Lactotransferrin |
| 534 | LY75 | Hs.153563 | 4065 | Lymphocyte antigen 75 |
| 535 | LY86 | Hs.653138 | 9450 | Lymphocyte antigen 86 |
| 536 | LY9 | Hs.403857 | 4063 | Lymphocyte antigen 9 |
| 537 | LYN | Hs.699154 | 4067 | V-yes-1 Yamaguchi sarcoma viral related oncogene homolog |
| 538 | LYST | Hs.532411 | 1130 | Lysosomal trafficking regulator |
| 539 | MADCAM1 | Hs.102598 | 8174 | Mucosal vascular addressin cell adhesion molecule 1 |
| 540 | MAFB | Hs.702085 | 9935 | V-maf musculoaponeurotic fibrosarcoma oncogene homolog B (avian) |
| 541 | MAG | Hs.348346 | 27307 | Malignancy-associated gene |
| 542 | MALT1 | Hs.601217 | 10892 | Mucosa associated lymphoid tissue lymphoma translocation gene 1 |
| 543 | MAP3K7 | Hs.644143 | 6885 | Mitogen-activated protein kinase kinase kinase 7 |
| 544 | MAP4K2 | Hs.534341 | 5871 | Mitogen-activated protein kinase kinase kinase kinase 2 |
| 545 | MASP1 | Hs.89983 | 5648 | Mannan-binding lectin serine peptidase 1 (C4/C2 activating component of Ra-reactive factor) |
| 546 | MASP2 | Hs.655645 | 10747 | Mannan-binding lectin serine peptidase 2 |
| 547 | MBL2 | Hs.499674 | 4153 | Mannose-binding lectin (protein C) 2, soluble (opsonic defect) |
| 548 | MBP | Hs.551713 | 4155 | Myelin basic protein |
| 549 | MEIS3P1 | Hs.356135 | 4213 | Meis homeobox 3 pseudogene 1 |
| 550 | MET | Hs.132966 | 4233 | Met proto-oncogene (hepatocyte growth factor receptor) |
| 551 | MIA3 | Hs.118474 | 375056 | Melanoma inhibitory activity family, member 3 |
| 552 | MICA | Hs.549053 | 4276 | MHC class I polypeptide-related sequence A |
| 553 | MICB | Hs.211580 | 4277 | MHC class I polypeptide-related sequence B |
| 554 | MIF | Hs.407995 | 4282 | Macrophage migration inhibitory factor (glycosylation-inhibiting factor) |
| 555 | MLF1 | Hs.85195 | 4291 | Myeloid leukemia factor 1 |
| 556 | MLL | Hs.258855 | 4297 | Myeloid/lymphoid or mixed-lineage leukemia (trithorax homolog, Drosophila) |
| 557 | MMP7 | Hs.2256 | 4316 | Matrix metallopeptidase 7 (matrilysin, uterine) |
| 558 | MMP9 | Hs.297413 | 4318 | Matrix metallopeptidase 9 (gelatinase B, 92kDa gelatinase, 92kDa type IV collagenase) |
| 559 | MNX1 | Hs.37035 | 3110 | Motor neuron and pancreas homeobox 1 |
| 560 | MR1 | Hs.101840 | 3140 | Major histocompatibility complex, class I-related |
| 561 | MS4A1 | Hs.438040 | 931 | Membrane-spanning 4-domains, subfamily A, member 1 |
| 562 | MS4A2 | Hs.386748 | 2206 | Membrane-spanning 4-domains, subfamily A, member 2 (Fc fragment of IgE, high affinity I, receptor f |
| 563 | MSH2 | Hs.597656 | 4436 | MutS homolog 2, colon cancer, nonpolyposis type 1 (E. coli) |
| 564 | MSN | Hs.87752 | 4478 | Moesin |
| 565 | MSR1 | Hs.147635 | 4481 | Macrophage scavenger receptor 1 |
| 566 | MYD88 | Hs.82116 | 4615 | Myeloid differentiation primary response gene (88) |
| 567 | MYH9 | Hs.474751 | 4627 | Myosin, heavy chain 9, non-muscle |
| 568 | MYST1 | Hs.533803 | 84148 | MYST histone acetyltransferase 1 |
| 569 | MYST3 | Hs.491577 | 7994 | MYST histone acetyltransferase (monocytic leukemia) 3 |
| 570 | NCAM1 | Hs.503878 | 4684 | Neural cell adhesion molecule 1 |
| 571 | NCF1C | Hs.648940 | 654817 | Neutrophil cytosolic factor 1C pseudogene |
| 572 | NCF2 | Hs.587558 | 4688 | Neutrophil cytosolic factor 2 (65kDa, chronic granulomatous disease, autosomal 2) |
| 573 | NCF4 | Hs.474781 | 4689 | Neutrophil cytosolic factor 4, 40kDa |
| 574 | NCK1 | Hs.477693 | 4690 | NCK adaptor protein 1 |
| 575 | NCK2 | Hs.695241 | 8440 | NCK adaptor protein 2 |
| 576 | NCOA6 | Hs.368971 | 23054 | Nuclear receptor coactivator 6 |
| 577 | NCR1 | Hs.97084 | 9437 | Natural cytotoxicity triggering receptor 1 |
| 578 | NCR2 | Hs.194721 | 9436 | Natural cytotoxicity triggering receptor 2 |
| 579 | NCR3 | Hs.509513 | 259197 | Natural cytotoxicity triggering receptor 3 |

| | | | | |
|---|---|---|---|---|
| 580 | NFIL3 | Hs.79334 | 4783 | Nuclear factor, interleukin 3 regulated |
| 581 | NGF | Hs.2561 | 4803 | Nerve growth factor (beta polypeptide) |
| 582 | NGFR | Hs.415768 | 4804 | Nerve growth factor receptor (TNFR superfamily, member 16) |
| 583 | NHEJ1 | Hs.225988 | 79840 | Nonhomologous end-joining factor 1 |
| 584 | NKX2-5 | Hs.54473 | 1482 | NK2 transcription factor related, locus 5 (Drosophila) |
| 585 | NOD2 | Hs.592072 | 64127 | Nucleotide-binding oligomerization domain containing 2 |
| 586 | NOS1 | Hs.654410 | 4842 | Nitric oxide synthase 1 (neuronal) |
| 587 | NOS2 | Hs.709191 | 4843 | Nitric oxide synthase 2, inductible |
| 588 | NOS3 | Hs.699165 | 4846 | Nitric oxide synthase 3 (endothelial cell) |
| 589 | NOTCH1 | Hs.495473 | 4851 | Notch homolog 1, translocation-associated (Drosophila) |
| 590 | NOTCH2 | Hs.487360 | 4853 | Notch homolog 2 (Drosophila) |
| 591 | NOTCH4 | Hs.436100 | 4855 | Notch homolog 4 (Drosophila) |
| 592 | NPM1 | Hs.557550 | 4869 | Nucleophosmin (nucleolar phosphoprotein B23, numatrin) |
| 593 | NPTN | Hs.187866 | 27020 | Neuroplastin |
| 594 | ODZ1 | Hs.23796 | 10178 | Odz, odd Oz/ten-m homolog 1(Drosophila) |
| 595 | OPRD1 | Hs.372 | 4985 | Opioid receptor, delta 1 |
| 596 | OPRK1 | Hs.106795 | 4986 | Opioid receptor, kappa 1 |
| 597 | PAX5 | Hs.654464 | 5079 | Paired box 5 |
| 598 | PDCD1 | Hs.158297 | 5133 | Programmed cell death 1 |
| 599 | PDCD1LG2 | Hs.532279 | 80380 | Programmed cell death 1 ligand 2 |
| 600 | PDGFA | Hs.705381 | 5154 | Platelet-derived growth factor alpha polypeptide |
| 601 | PDGFB | Hs.1976 | 5155 | Platelet-derived growth factor beta polypeptide (simian sarcoma viral (v-sis) oncogene homolog) |
| 602 | PDGFC | Hs.570855 | 56034 | Platelet derived growth factor C |
| 603 | PDGFD | Hs.352298 | 80310 | Platelet derived growth factor D |
| 604 | PDGFRA | Hs.74615 | 5156 | Platelet-derived growth factor receptor, alpha polypeptide |
| 605 | PDGFRB | Hs.509067 | 5159 | Platelet-derived growth factor receptor, beta polypeptide |
| 606 | PF4 | Hs.81564 | 5196 | Platelet factor 4 (chemokine (C-X-C motif) ligand 4) |
| 607 | PF4V1 | Hs.72933 | 5197 | Platelet factor 4 variant 1 |
| 608 | PGF | Hs.252820 | 5228 | Placental growth factor, vascular endothelial growth factor-related protein |
| 609 | PGLYRP1 | Hs.137583 | 8993 | Peptidoglycan recognition protein 1 |
| 610 | PGLYRP4 | Hs.58356 | 57115 | Peptidoglycan recognition protein 4 |
| 611 | PGRMC1 | Hs.90061 | 10857 | Progesterone receptor membrane component 1 |
| 612 | PLA2G1B | Hs.992 | 5319 | Phospholipase A2, group IB (pancreas) |
| 613 | PLUNC | Hs.211092 | 51297 | Palate, lung and nasal epithelium carcinoma associated |
| 614 | PMF1 | Hs.530479 | 11243 | Polyamine-modulated factor 1 |
| 615 | PML | Hs.526464 | 5371 | Promyelocytic leukemia |
| 616 | PNP | Hs.75514 | 4860 | Purine nucleoside phosphorylase |
| 617 | POLL | Hs.523230 | 27343 | Polymerase (DNA directed), lambda |
| 618 | POU2AF1 | Hs.654525 | 5450 | POU class 2 associating factor 1 |
| 619 | POU2F2 | Hs.654420 | 5452 | POU class 2 homeobox 2 |
| 620 | PPARG | Hs.162646 | 5468 | Peroxisome proliferator-activated receptor gamma |
| 621 | PPBP | Hs.2164 | 5473 | Pro-platelet basic protein (chemokine (C-X-C motif) ligand 7) |
| 622 | PRDX3 | Hs.523302 | 10935 | Peroxiredoxin 3 |
| 623 | PRF1 | Hs.2200 | 5551 | Perforin 1 (pore forming protein) |
| 624 | PRG3 | Hs.251386 | 10394 | Proteoglycan 3 |
| 625 | PRKRA | Hs.632540 | 8575 | Protein kinase, interferon-inducible double stranded RNA dependent activator |
| 626 | PRLR | Hs.368587 | 5618 | Prolactin receptor |
| 627 | PROS1 | Hs.64016 | 5627 | Protein S (alpha) |
| 628 | PSMB10 | Hs.9661 | 5699 | Proteasome (prosome, macropain) subunit, beta type, 10 |
| 629 | PTAFR | Hs.77542 | 5724 | Platelet-activating factor receptor |
| 630 | PTCRA | Hs.169002 | 171558 | Pre T-cell antigen receptor alpha |
| 631 | PTGER4 | Hs.199248 | 5734 | Prostaglandin E receptor 4 (subtype EP4) |
| 632 | PTGS2 | Hs.196384 | 5743 | Prostaglandin-endoperoxide synthase 2 (prostaglandin G/H synthase and cyclooxygenase) |
| 633 | PTPRC | Hs.654514 | 5788 | Protein tyrosine phosphatase, receptor type, C |
| 634 | PVR | Hs.171844 | 5817 | Poliovirus receptor |
| 635 | PVRL1 | Hs.334846 | 5818 | Poliovirus receptor-related 1 (herpesvirus entry mediator C) |
| 636 | PVRL2 | Hs.655455 | 5819 | Poliovirus receptor-related 2 (herpesvirus entry mediator B) |
| 637 | PXDN | Hs.332197 | 7837 | Peroxidasin homolog (Drosophila) |

| | | | | |
|---|---|---|---|---|
| 638 | RAG1 | Hs.73958 | 5896 | Recombination activating gene 1 |
| 639 | RAG2 | Hs.159376 | 5897 | Recombination activating gene 2 |
| 640 | RARA | Hs.654583 | 5914 | Retinoic acid receptor, alpha |
| 641 | RBP4 | Hs.50223 | 5950 | Retinol binding protein 4, plasma |
| 642 | RELA | Hs.502875 | 5970 | V-rel reticuloendotheliosis viral oncogene homolog A, nuclear factor of kappa light polypeptide gene e |
| 643 | RFX1 | Hs.655215 | 5989 | Regulatory factor X, 1 (influences HLA class II expression) |
| 644 | RGS1 | Hs.75256 | 5996 | Regulator of G-protein signaling 1 |
| 645 | RHOH | Hs.654594 | 399 | Ras homolog gene family, member H |
| 646 | ROCK1 | Hs.306307 | 6093 | Rho-associated, coiled-coil containing protein kinase 1 |
| 647 | RPS14 | Hs.381126 | 6208 | Ribosomal protein S14 |
| 648 | RPS19 | Hs.438429 | 6223 | Ribosomal protein S19 |
| 649 | RSAD2 | Hs.17518 | 91543 | Radical S-adenosyl methionine domain containing 2 |
| 650 | RTEL1 | Hs.730810 | 51750 | Regulator of telomere elongation helicase 1 |
| 651 | RUNX1 | Hs.149261 | 861 | Runt-related transcription factor 1 (acute myeloid leukemia 1; aml1 oncogene) |
| 652 | S100A7 | Hs.112408 | 6278 | S100 calcium binding protein A7 |
| 653 | S100A8 | Hs.416073 | 6279 | S100 calcium binding protein A8 |
| 654 | S100A9 | Hs.112405 | 6280 | S100 calcium binding protein A9 |
| 655 | S1PR4 | Hs.662006 | 8698 | Sphingosine-1-phosphate receptor 4 |
| 656 | SAMHD1 | Hs.580681 | 25939 | SAM domain and HD domain 1 |
| 657 | SART1 | Hs.502883 | 9092 | Squamous cell carcinoma antigen recognized by T cells |
| 658 | SBNO2 | Hs.408708 | 22904 | Strawberry notch homolog 2 (Drosophila) |
| 659 | SCARB2 | Hs.349656 | 950 | Scavenger receptor class B, member 2 |
| 660 | SCG2 | Hs.516726 | 7857 | Secretogranin II (chromogranin C) |
| 661 | SCIN | Hs.655515 | 85477 | Scinderin |
| 662 | SDF2 | Hs.514036 | 6388 | Stromal cell-derived factor 2 |
| 663 | SDF4 | Hs.42806 | 51150 | Stromal cell derived factor 4 |
| 664 | SECTM1 | Hs.558009 | 6398 | Secreted and transmembrane 1 |
| 665 | SEMA3C | Hs.269109 | 10512 | Sema domain, immunoglobulin domain (Ig), short basic domain, secreted, (semaphorin) 3C |
| 666 | SEMA4D | Hs.655281 | 10507 | Sema domain, immunoglobulin domain (Ig), transmembrane domain (TM) and short cytoplasmic dom |
| 667 | SEMA7A | Hs.24640 | 8482 | Semaphorin 7A, GPI membrane anchor (John Milton Hagen blood group) |
| 668 | SERPINB3 | Hs.227948 | 6317 | Serpin peptidase inhibitor, clade B (ovalbumin), member 3 |
| 669 | SERPINB4 | Hs.123035 | 6318 | Serpin peptidase inhibitor, clade B (ovalbumin), member 4 |
| 670 | SFTPD | Hs.253495 | 6441 | Surfactant, pulmonary-associated protein D |
| 671 | SIGLEC1 | Hs.31869 | 6614 | Sialic acid binding Ig-like lectin 1, sialoadhesin |
| 672 | SIGLEC5 | Hs.310333 | 8778 | Sialic acid binding Ig-like lectin 5 |
| 673 | SIGLEC6 | Hs.397255 | 946 | Sialic acid binding Ig-like lectin 6 |
| 674 | SIGLEC7 | Hs.699304 | 27036 | Sialic acid binding Ig-like lectin 7 |
| 675 | SIGLEC8 | Hs.447899 | 27181 | Sialic acid binding Ig-like lectin 8 |
| 676 | SIGLEC9 | Hs.245828 | 27180 | Sialic acid binding Ig-like lectin 9 |
| 677 | SIRPG | Hs.590883 | 55423 | Signal-regulatory protein gamma |
| 678 | SIT1 | Hs.88012 | 27240 | Signaling threshold regulating transmembrane adaptor 1 |
| 679 | SIVA1 | Hs.112058 | 10572 | SIVA1, apoptosis-inducing factor |
| 680 | SKAP1 | Hs.316931 | 8631 | Src kinase associated phosphoprotein 1 |
| 681 | SLAMF7 | Hs.517265 | 57823 | SLAM family member 7 |
| 682 | SLC11A1 | Hs.591607 | 6556 | Solute carrier family 11 (proton-coupled divalent metal ion transporters), member 1 |
| 683 | SMAD3 | Hs.618504 | 4088 | SMAD family member 3 |
| 684 | SMAD6 | Hs.153863 | 4091 | SMAD family member 6 |
| 685 | SNRK | Hs.476052 | 54861 | SNF related kinase |
| 686 | SOCS5 | Hs.468426 | 9655 | Suppressor of cytokine signaling 5 |
| 687 | SOD1 | Hs.443914 | 6647 | Superoxide dismutase 1, soluble (amyotrophic lateral sclerosis 1 (adult)) |
| 688 | SOX4 | Hs.699195 | 6659 | SRY (sex determining region Y)-box 4 |
| 689 | SP100 | Hs.369056 | 6672 | SP100 nuclear antigen |
| 690 | SP2 | Hs.514276 | 6668 | Sp2 transcription factor |
| 691 | SPG21 | Hs.242458 | 51324 | Spastic paraplegia 21 (autosomal recessive, Mast syndrome) |
| 692 | SPI1 | Hs.502511 | 6688 | Spleen focus forming virus (SFFV) proviral integration oncogene spi1 |
| 693 | SPINK5 | Hs.331555 | 11005 | Serine peptidase inhibitor, Kazal type 5 |
| 694 | ST6GAL1 | Hs.207459 | 6480 | ST6 beta-galactosamide alpha-2,6-sialyltranferase 1 |
| 695 | SYK | Hs.371720 | 6850 | Spleen tyrosine kinase |

| | | | | |
|---|---|---|---|---|
| 696 | TAP1 | Hs.352018 | 6890 | Transporter 1, ATP-binding cassette, sub-family B (MDR/TAP) |
| 697 | TAP2 | Hs.502 | 6891 | Transporter 2, ATP-binding cassette, sub-family B (MDR/TAP) |
| 698 | TAPBP | Hs.370937 | 6892 | TAP binding protein (tapasin) |
| 699 | TARBP2 | Hs.326 | 6895 | TAR (HIV-1) RNA binding protein 2 |
| 700 | TAZ | Hs.409911 | 6901 | Tafazzin (cardiomyopathy, dilated 3A (X-linked); endocardial fibroelastosis 2; Barth syndrome) |
| 701 | TBX1 | Hs.173984 | 6899 | T-box 1 |
| 702 | TBX21 | Hs.272409 | 30009 | T-box 21 |
| 703 | TCF12 | Hs.511504 | 6938 | Transcription factor 12 (HTF4, helix-loop-helix transcription factors 4) |
| 704 | TCF3 | Hs.371282 | 6929 | Transcription factor 3 (E2A immunoglobulin enhancer binding factors E12/E47) |
| 705 | TCF7 | Hs.573153 | 6932 | Transcription factor 7 (T-cell specific, HMG-box) |
| 706 | TGFA | Hs.170009 | 7039 | Transforming growth factor, alpha |
| 707 | TGFB1 | Hs.645227 | 7040 | Transforming growth factor, beta 1 |
| 708 | TGFB2 | Hs.133379 | 7042 | Transforming growth factor, beta 2 |
| 709 | TGFB3 | Hs.592317 | 7043 | Transforming growth factor, beta 3 |
| 710 | TGFBR1 | Hs.494622 | 7046 | Transforming growth factor, beta receptor I (activin A receptor type II-like kinase, 53kDa) |
| 711 | TGFBR2 | Hs.82028 | 7048 | Transforming growth factor, beta receptor II (70/80kDa) |
| 712 | TGFBR3 | Hs.482390 | 7049 | Transforming growth factor, beta receptor III |
| 713 | THBS1 | Hs.164226 | 7057 | Thrombospondin 1 |
| 714 | THY1 | Hs.653181 | 7070 | Thy-1 cell surface antigen |
| 715 | TLR1 | Hs.621817 | 7096 | Toll-like receptor 1 |
| 716 | TLR2 | Hs.519033 | 7097 | Toll-like receptor 2 |
| 717 | TLR3 | Hs.657724 | 7098 | Toll-like receptor 3 |
| 718 | TLR4 | Hs.174312 | 7099 | Toll-like receptor 4 |
| 719 | TLR5 | Hs.604542 | 7100 | Toll-like receptor 5 |
| 720 | TLR6 | Hs.662185 | 10333 | Toll-like receptor 6 |
| 721 | TLR7 | Hs.659215 | 51284 | Toll-like receptor 7 |
| 722 | TLR8 | Hs.660543 | 51311 | Toll-like receptor 8 |
| 723 | TM7SF4 | Hs.652230 | 81501 | Transmembrane 7 superfamily member 4 |
| 724 | TMX1 | Hs.125221 | 81542 | Thioredoxin-related transmembrane protein 1 |
| 725 | TNF | Hs.241570 | 7124 | Tumor necrosis factor (TNF superfamily, member 2) |
| 726 | TNFAIP1 | Hs.76090 | 7126 | Tumor necrosis factor, alpha-induced protein 1 (endothelial) |
| 727 | TNFRSF10B | Hs.521456 | 8795 | Tumor necrosis factor receptor superfamily, member 10b |
| 728 | TNFRSF10C | Hs.655801 | 8794 | Tumor necrosis factor receptor superfamily, member 10c, decoy without an intracellular domain |
| 729 | TNFRSF10D | Hs.213467 | 8793 | Tumor necrosis factor receptor superfamily, member 10d, decoy with truncated death domain |
| 730 | TNFRSF11A | Hs.204044 | 8792 | Tumor necrosis factor receptor superfamily, member 11a, NFKB activator |
| 731 | TNFRSF11B | Hs.81791 | 4982 | Tumor necrosis factor receptor superfamily, member 11b (osteoprotegerin) |
| 732 | TNFRSF12A | Hs.355899 | 51330 | Tumor necrosis factor receptor superfamily, member 12A |
| 733 | TNFRSF13B | Hs.158341 | 23495 | Tumor necrosis factor receptor superfamily, member 13B |
| 734 | TNFRSF14 | Hs.512898 | 8764 | Tumor necrosis factor receptor superfamily, member 14 (herpesvirus entry mediator) |
| 735 | TNFRSF17 | Hs.2556 | 608 | Tumor necrosis factor receptor superfamily, member 17 |
| 736 | TNFRSF1A | Hs.279594 | 7132 | Tumor necrosis factor receptor superfamily, member 1A |
| 737 | TNFRSF1B | Hs.256278 | 7133 | Tumor necrosis factor receptor superfamily, member 1B |
| 738 | TNFRSF21 | Hs.443577 | 27242 | Tumor necrosis factor receptor superfamily, member 21 |
| 739 | TNFRSF25 | Hs.462529 | 8718 | Tumor necrosis factor receptor superfamily, member 25 |
| 740 | TNFRSF4 | Hs.129780 | 7293 | Tumor necrosis factor receptor superfamily, member 4 |
| 741 | TNFRSF8 | Hs.1314 | 943 | Tumor necrosis factor receptor superfamily, member 8 |
| 742 | TNFRSF9 | Hs.654459 | 3604 | Tumor necrosis factor receptor superfamily, member 9 |
| 743 | TNFSF10 | Hs.478275 | 8743 | Tumor necrosis factor (ligand) superfamily, member 10 |
| 744 | TNFSF11 | Hs.333791 | 8600 | Tumor necrosis factor (ligand) superfamily, member 11 |
| 745 | TNFSF12 | Hs.54673 | 8742 | Tumor necrosis factor (ligand) superfamily, member 12 |
| 746 | TNFSF13 | Hs.54673 | 8741 | Tumor necrosis factor (ligand) superfamily, member 13 |
| 747 | TNFSF14 | Hs.129708 | 8740 | Tumor necrosis factor (ligand) superfamily, member 14 |
| 748 | TNFSF15 | Hs.241382 | 9966 | Tumor necrosis factor (ligand) superfamily, member 15 |
| 749 | TNFSF18 | Hs.248197 | 8995 | Tumor necrosis factor (ligand) superfamily, member 18 |
| 750 | TNFSF4 | Hs.181097 | 7292 | Tumor necrosis factor (ligand) superfamily, member 4 (tax-transcriptionally activated glycoprotein 1, 3 |
| 751 | TNFSF8 | Hs.654445 | 944 | Tumor necrosis factor (ligand) superfamily, member 8 |
| 752 | TNFSF9 | Hs.1524 | 8744 | Tumor necrosis factor (ligand) superfamily, member 9 |
| 753 | TOLLIP | Hs.368527 | 54472 | Toll interacting protein |

| | | | | |
|---|---|---|---|---|
| 754 | TPD52 | Hs.368433 | 7163 | Tumor protein D52 |
| 755 | TRAF2 | Hs.522506 | 7186 | TNF receptor-associated factor 2 |
| 756 | TRAF6 | Hs.591983 | 7189 | TNF receptor-associated factor 6 |
| 757 | TRAT1 | Hs.138701 | 50852 | T cell receptor associated transmembrane adaptor 1 |
| 758 | TRBC1 | - | 28639 | T cell receptor beta constant 1 |
| 759 | TREM1 | Hs.283022 | 54210 | Triggering receptor expressed on myeloid cells 1 |
| 760 | TREM2 | Hs.435295 | 54209 | Triggering receptor expressed on myeloid cells 2 |
| 761 | TRGV5 | - | 6978 | T cell receptor gamma variable 5 |
| 762 | TRIM10 | Hs.274295 | 10107 | Tripartite motif-containing 10 |
| 763 | TRIM22 | Hs.501778 | 10346 | Tripartite motif-containing 22 |
| 764 | TRPC4AP | Hs.168073 | 26133 | Transient receptor potential cation channel, subfamily C, member 4 associated protein |
| 765 | TUBB | Hs.636480 | 203068 | Tubulin, beta |
| 766 | TUBB2C | Hs.433615 | 10383 | Tubulin, beta 2C |
| 767 | TUBB3 | Hs.511743 | 10381 | Tubulin, beta 3 |
| 768 | TXLNA | Hs.699306 | 200081 | Taxilin alpha |
| 769 | TYROBP | Hs.515369 | 7305 | TYRO protein tyrosine kinase binding protein |
| 770 | UBE2N | Hs.524630 | 7334 | Ubiquitin-conjugating enzyme E2N (UBC13 homolog, yeast) |
| 771 | ULBP2 | Hs.656778 | 80328 | UL16 binding protein 2 |
| 772 | VAMP7 | Hs.24167 | 6845 | Vesicle-associated membrane protein 7 |
| 773 | VCAM1 | Hs.109225 | 7412 | Vascular cell adhesion molecule 1 |
| 774 | VEGFA | Hs.73793 | 7422 | Vascular endothelial growth factor A |
| 775 | VEGFB | Hs.78781 | 7423 | Vascular endothelial growth factor B |
| 776 | VEGFC | Hs.435215 | 7424 | Vascular endothelial growth factor C |
| 777 | VIPR1 | Hs.348500 | 7433 | Vasoactive intestinal peptide receptor 1 |
| 778 | VPREB1 | Hs.247979 | 7441 | Pre-B lymphocyte gene 1 |
| 779 | VTCN1 | Hs.546434 | 79679 | V-set domain containing T cell activation inhibitor 1 |
| 780 | VTN | Hs.2257 | 7448 | Vitronectin |
| 781 | WAS | Hs.2157 | 7454 | Wiskott-Aldrich syndrome (eczema-thrombocytopenia) |
| 782 | XBP1 | Hs.437638 | 7494 | X-box binding protein 1 |
| 783 | XCL1 | Hs.546295 | 6375 | Chemokine (C motif) ligand 1 |
| 784 | XCR1 | Hs.248116 | 2829 | Chemokine (C motif) receptor 1 |
| 785 | YTHDF2 | Hs.532286 | 51441 | YTH domain family, member 2 |
| 786 | ZAP70 | Hs.234569 | 7535 | Zeta-chain (TCR) associated protein kinase 70kDa |
| 787 | ZBTB16 | Hs.591945 | 7704 | Zinc finger and BTB domain containing 16 |
| 788 | ZEB1 | Hs.124503 | 6935 | Zinc finger E-box binding homeobox 1 |
| 789 | ZNF160 | Hs.655967 | 90338 | Zinc finger protein 160 |
| 790 | ZNF3 | Hs.435302 | 7551 | Zinc finger protein 3 |
| 791 | ZNF675 | Hs.264345 | 171392 | Zinc finger protein 675 |

**Supplementary Table 1:** List of IA genes.

| Gene | Probe | p value Step-cox | p value quartile method | p value classical cox | 6-IA gene-Risk |
|---|---|---|---|---|---|
| **ACVR2A** | **205327_s_at** | **<1.0E-4** | **1.53E-4** | **1.3E-3** | **yes** |
| **ARG1** | **206177_s_at** | **<1.0E-4** | **1.99E-3** | | **yes** |
| **CD22** | **204581_at** | **<1.0E-4** | **<1.0E-4** | **3.8E-3** | **yes** |
| **FGF2** | **204422_s_at** | | **3.39E-3** | **4.9E-3** | **yes** |
| **MNX1** | **214614_at** | **<1.0E-4** | **4.88E-3** | **4.0E-4** | **yes** |
| **RPS19** | **213414_s_at** | | **3.87E-4** | | **yes** |
| ADAMDEC1 | 206134_at | <1.0E-4 | | | |
| ADIPOQ | 207175_at | <1.0E-4 | | | |
| BCL11A | 219497_s_at | | | 9.8E-4 | |
| BLNK | 207655_s_at | | | 3.7E-3 | |
| BMPR1A | 213578_at | | <1.0E-4 | | |
| BNIP3L | 221478_at | | 3.95E-3 | | |
| C1QBP | 214214_s_at | | 9.47E-3 | | |
| C5AR1 | 220088_at | <1.0E-4 | | | |
| CALCA | 217495_x_at | | 1.87E-3 | | |
| CARTPT | 206339_at | | 4.65E-4 | | |
| CCBP2 | 206887_at | | | 7.2E-3 | |
| CCL15 | 210390_s_at | 9.0E-4 | | | |
| CCL17 | 207900_at | <1.0E-4 | 5.79E-3 | | |
| CCR10 | 220565_at | <1.0E-4 | | | |
| CD19 | 206398_s_at | <1.0E-4 | | | |
| CD1E | 215784_at | <1.0E-4 | | | |
| CD247 | 210031_at | | 3.34E-3 | | |
| CD70 | 206508_at | | 1.86E-3 | | |
| CD79B | 205297_s_at | | 9.43E-3 | | |
| CDKN2A | 211156_at | <1.0E-4 | | | |
| CFP | 206380_s_at | | | 8.7E-3 | |
| CHIT1 | 208168_s_at | | | 4.4E-3 | |
| CLEC10A | 206682_at | 1.4E-3 | | | |
| CRHR1 | 214619_at | | 5.94E-4 | | |
| CSF1 | 209716_at; 207082_at; 211839_s_at | | | 1.6E-3 | |
| CSF1R | 203104_at | | | 1.7E-3 | |
| CSF2RA | 210340_s_at | | | 4.7E-3 | |
| CTSG | 205653_at | <1.0E-4 | | | |
| CX3CL1 | 203687_at; 823_at | | | 7.4E-4 | |
| CX3CR1 | 205898_at | | | 1.5E-3 | |
| CXADR | 203917_at | | | 7.7E-3 | |
| CXCL11 | 211122_s_at | 2.0E-3 | | | |
| CXCR4 | 217028_at | 2.0E-4 | | | |

| | | | | |
|---|---|---|---|---|
| DHRS2 | 214079_at | <1.0E-4 | | |
| EDNRB | 204271_s_at | | <1.0E-4 | 7.2E-3 |
| ERAP1 | 214012_at | | 6.89E-3 | |
| EXOSC9 | 205061_s_at | | | 6.0E-3 |
| CD89 | 211307_s_at | 6.0E-4 | | |
| FCGR1B/ C | 214511_x_at | | 9.67E-3 | |
| FCGRT | 218831_s_at | | | 8.1E-3 |
| FGF12 | 214589_at | | 8.49E-3 | |
| FGF17 | 221376_at | <1.0E-4 | | |
| FGFR2 | 208225_at | | | 3.6E-3 |
| FOXO3 | 217399_s_at | | | 9.0E-3 |
| FYN | 216033_s_at | <1.0E-4 | | |
| GPR183 | 205419_at | 3.62 E-3 | | |
| HDAC9 | 205659_at | <1.0E-4 | | |
| HGF | 210998_s_at | <1.0E-4 | | |
| HIF1A | 200989_at | | 1.46E-3 | |
| HMGB1 | 200679_x_at | | 9.37E-3 | |
| HSP90AA1 | 211969_at | <1.0E-4 | | |
| ICOSLG | 213450_s_at | <1.0E-4 | | |
| IGHD/IGHG1 | 215621_s_at | <1.0E-4 | | |
| IGLL1 | 213502_x_at | <1.0E-4 | | |
| IGSF6 | 206420_at | | 6.12E-3 | |
| IKBKG | 36004_at | | 8.49E-3 | |
| IL1B | 205067_at | | | 5.1E-3 |
| IL21R | 221658_s_at | | 3.89E-3 | 7.6E-3 |
| IL33 | 209821_at | | 9.55E-3 | |
| IL4 | 207539_s_at | <1.0E-4 | | |
| IL5 | 207952_at | <1.0E-4 | 5.60E-3 | |
| IL6ST | 204863_s_at | | 3.77E-3 | 7.2E-3 |
| INHBA | 210511_s_at | 4.0E-4 | | |
| JAG2 | 209784_s_at | | 5.33E-3 | |
| KIR2DL1 | 208179_x_at | <1.0E-4 | | |
| KIR2DL4 | 208426_x_at | <1.0E-4 | | |
| KIR3DL3 | 216676_x_at | <1.0E-4 | | |
| LDB1 | 35160_at | | 5.53E-3 | |
| LTB4R | 216388_s_at | <1.0E-4 | | |
| LYN | 210754_s_at; 202625_at | | | 4.6E-3 |
| MAP4K2 | 204936_at | | <1.0E-4 | |
| MET | 203510_at | | 4.22E-3 | |
| MS4A1 | 217418_x_at | | 6.27E-3 | |
| MYST3 | 216361_s_at | <1.0E-4 | | |
| NCF1B | 214084_x_at | <1.0E-4 | | |
| NCK1 | 211063_s_at | <1.0E-4 | <1.0E-4 | |
| NOS2A | 210037_s_at | <1.0E-4 | | |
| PDGFC | 218718_at | | 1.14E-3 | 2.3E-3 |

| | | | | |
|---|---|---|---|---|
| PML | 211013_x_at | 2.45E-3 | 1.92E-3 | |
| POU2F2 | 211771_s_at | <1.0E-4 | | |
| PPARG | 208510_s_at | <1.0E-4 | | |
| PTCRA | 211837_s_at | <1.0E-4 | | |
| RBP4 | 219140_s_at | | 2.78E-3 | |
| RELA | 209878_s_at | | | 7.4E-3 |
| RPS14 | 208645_s_at | | 7.73E-3 | |
| S1PR4 | 206437_at | <1.0E-4 | | |
| SCYE1 | 202541_at | | 2.53E-3 | |
| SERPINB4 | 211906_s_at | <1.0E-4 | | |
| SMAD3 | 218284_at | | | 3.9E-3 |
| TAPBP | 210294_at | | 2.54E-3 | |
| TGFB1 | 203084_at | | 6.74E-3 | |
| TM7SF4 | 221266_s_at | <1.0E-4 | | |
| TNFRSF25 | 211841_s_at | | 1.65E-4 | |
| TNFRSF6B | 206092_x_at | | 5.32E-3 | |
| TNFSF12/ 13 | 205611_at | | 8.34E-3 | 8.6E-3 |
| TPD52 | 201688_s_at | <1.0E-4 | | |
| TRIM10 | 210579_s_at | <1.0E-4 | | |
| TUBB3/2C | 202154_x_at | <1.0E-4 | | |
| ULBP2 | 221291_at | <1.0E-4 | 2.44E-3 | |
| VEGFC | 209946_at | <1.0E-4 | | |
| VTCN1 | 219768_at | <1.0E-4 | | |
| ZEB1 | 212758_s_at | | <1.0E-4 | |

**Supplementary Table 2:** IA genes associated with survival in the 3 statistical methods.
